# The EORTC QLU-C10D discrete choice experiment for cancer patients: a first step towards patient utility weights

**DOI:** 10.1186/s41687-022-00430-5

**Published:** 2022-05-04

**Authors:** Eva-Maria Gamper, Madeleine T. King, Richard Norman, Fanny L. C. Loth, Bernhard Holzner, Georg Kemmler, the EORTC Quality of Life Group^

**Affiliations:** 1 Innsbruck Institute of Patient-Centered Outcome Research (IIPCOR), Dr. Stumpf Straße 47, 6020 Innsbruck, Austria; 2 School of Psychology, Faculty of Science, University of Sydney, Sydney, NSW 2006 Australia; 3 School of Public Health, Curtin University, Perth, Australia; 4 Faculty of Philosophy and Education, Catholic University of Eichstätt-Ingolstadt, Eichtstätt, Germany; 5 Department of Psychiatry, Psychotherapy and Psychosomatics, Psychiatry II, Medical University of Innsbruck, Innsbruck, Austria; 6 Department of Psychiatry, Psychotherapy and Psychosomatics, Psychiatry I, Medical University of Innsbruck, Innsbruck, Austria

**Keywords:** QLU-C10D, Utility instrument, Patient utilities, Discrete choice experiment, Trade-off, Quality of life

## Abstract

**Background:**

The European Organisation for Research and Treatment of Cancer (EORTC) Quality of Life Utility-Core 10 Dimensions (QLU-C10D) is a novel cancer-specific preference-based measure (PBM) for which value sets are being developed for an increasing number of countries. This is done by obtaining health preferences from the respective general population. There is an ongoing discussion if instead patients suffering from the disease in question should be asked for their preferences. We used the QLU-C10D valuation survey, originally designed for use in the general population, in a sample of cancer patients in Austria to assess the methodology’s acceptability and applicability in this target group before obtaining QLU-C10D patient preferences.

**Methods:**

The core of the QLU-C10D valuation survey is a discrete choice experiment in which respondents are asked to give preferences for certain health states (described by a relatively large number of 10 quality of life domains) and an associated survival time. They therewith are asked to trade off quality of life against life time. As this might be a very burdensome task for cancer patients undergoing treatment, a cognitive interview was conducted in a pilot sample to assess burden and potential additional needs for explanation in order to be able to use the DCE for the development of QLU-C10D patient preferences. In addition, responses to general feedback questions on the survey were compared against responses from a matched control group from the already completed Austrian general population valuation survey.

**Results:**

We included 48 patients (mean age 59.9 years; 46% female). In the cognitive interview, the majority indicated that their experience with the survey was positive (85%) and overall clarity as good (90%). In response to the general feedback questions, patients rated the presentation of the health states less clear than matched controls (*p* = 0.008). There was no difference between patients and the general population concerning the difficulty in choosing between the health states (*p* = 0.344).

**Conclusion:**

Despite the relatively large number of DCE domains the survey was manageable for patients and allows going on with the QLU-C10D patient valuation study.

**Supplementary Information:**

The online version contains supplementary material available at 10.1186/s41687-022-00430-5.

## Introduction

Health utilities are a core parameter in health economic evaluations. They represent the “value” a specific population assigns to certain health states and are anchored at 1 (representing full/best imaginable health) and 0 (representing being dead). Negative values are possible for health states considered to be worse than dead. These values are used to adjust survival time for the quality of life that time is spent in. The respective outcome parameter in economic studies that combines survival time with quality of life (i.e. health utility) is the so-called Quality-Adjusted Life Year (QALY) [1, 2].

One broadly applied method of obtaining health utilities is the use of preference-based measures (PBMs). A PBM consists of two elements:

1. A health state description system (several dimensions of health/health-related quality of life (HRQOL) with different levels of impairment) which is administered like a questionnaire; and
2. Utility weights for each health state described by that health state description system.

Utility weights are developed in valuation studies in which representatives of a certain perspective on health, disease and treatment provide their preferences for sets of health states comprising different levels of functional status and symptom burden. There is, though, some controversy which perspective ought to be considered—the one of patients suffering from the disease being investigated and being familiar with related impairments or the one of the general population representing the tax payers’ perspective [3–5]. The main argument for the latter is that in a publicly funded health system with the main aim of maximizing health for society the tax payers’ perspective is imperative [6]. The opposing view emphasises the importance of having experienced health states before being able to value them, which on the downside, bears the risk of bias through adaptation as well as potential personal benefit [7]. There is some agreement that patient preferences are relevant in the context of clinical decision making [6, 8] while decision making in the context of health resource allocation requires general population preferences [7].

Related to this issue is the discussion concerning the relevance of disease-specificity of the health states that need to be valued. Most PBMs, such as the widely used EuroQoL 5-Dimensions (EQ-5D) [9], are generic, i.e. their health state description systems do not depict a specific condition and therefore allow obtained utilities to be used for comparisons across diseases. For certain medical conditions, generic PBMs may not be sufficiently relevant or sensitive [10, 11].

In the field of oncology, the availability of the cancer-specific PBM European Organisation for Research and Treatment of Cancer (EORTC) Quality of Life Utility—Core 10 Dimensions (QLU-C10D) [12] facilitates the generation of cancer-specific utilities. It is based on the widely used EORTC Quality of Life Questionnaire Core 30 (QLQ-C30) and comprises 13 of the 30 questions of the parent instrument representing 10 HRQOL domains (physical, role, social, and emotional functioning, pain, fatigue, insomnia, appetite loss, nausea, and bowel problems).

Valuation studies are currently being performed in various countries in a concerted approach of two international research initiatives, the Multi-Attribute Utility Cancer (MAUCa) Consortium and the EORTC Quality of Life Group (QLG), using a standardised methodology. In line with the mission statement of the EORTC QLG to increase the incorporation of the patient’s perspective into outcome assessment in oncology, we aim to obtain QLU-C10D utilities not only from the general population but also from cancer patients themselves.

For QLU-C10 valuations a standardised methodology is in place. Preferences are obtained using a discrete choice experiment (DCE) in which respondents are asked to make choices between (hypothetical) health scenarios described by the 10 QLU-C10D domains and different survival times. The DCE has proven to produce reliable results in general population samples [13–15] and QLU-C10D values sets so far have been completed for Australia [16], Germany [17], Austria, Italy, and Poland [18], France [19], UK [20] and Canada [21]. The number of 10 domains results in quite complex DCE tasks in which respondent have to consider a range of health issues and weigh these against a survival time. Considering the complexity of the tasks and cancer patients’ compromised health states and difficult personal situations, dealing with trade-offs between HRQOL and hypothetical survival times in the DCE may be emotionally very burdensome and cognitively more challenging for them than for the general population respondents.

Therefore, before obtaining patient preferences for the QLU-C10D we aim at investigation the developed valuation methodology for its acceptability and applicability in cancer patients. We investigated the DCE-survey in a pilot sample of cancer patients using a mixed-methods approach. We performed this study in Austrian patients since the QLU-C10D patient valuations likewise will be performed in Austria (Austrian general population valuation already has been completed [18]).

## Respondents and methods

### Patient sample

Cancer patients were recruited in 2017 at the Medical University of Innsbruck, Austria. We aimed to include patients of different age, diagnosis groups, and treatment modalities.

Eligibility criteria for patients were a diagnosis of cancer, age > 18, sufficient command of German, no overt cognitive impairments, and written informed consent. Clinical data was gathered from medical charts comprising information on the diagnosis (ICD-10), treatment approach (curative/palliative), previous and current treatment modalities (e.g. surgery, chemotherapy, or radiotherapy), current medication, and comorbidities (e.g. heart problems, arthritis/rheumatism, or asthma). Sociodemographic information was collected as part of the valuation survey (see below).

All respondents provided written informed consent. Ethical approval was obtained from the Medical University of Innsbruck [AN215-0016]. All patients who agreed to participation in the QLU-C10D valuation survey completed the entire interview. Survey completion and cognitive interviews took 30–45 min.

### Matched general population controls

The general population control group was drawn from the QLU-C10D Austrian general population valuation which has been performed in 2017 [22]. Recruitment and assessment were performed by Survey Engine (www.surveyengine.com), a company specialized in the web-based conduction of DCEs using internet panels. For the present study, we matched a control group from the 1000 Austrian general population respondents to the patient sample according to age, sex, and education to obtain a case control ratio of 1:4.

### QLU-C10D valuation survey

**Fig. 1:**
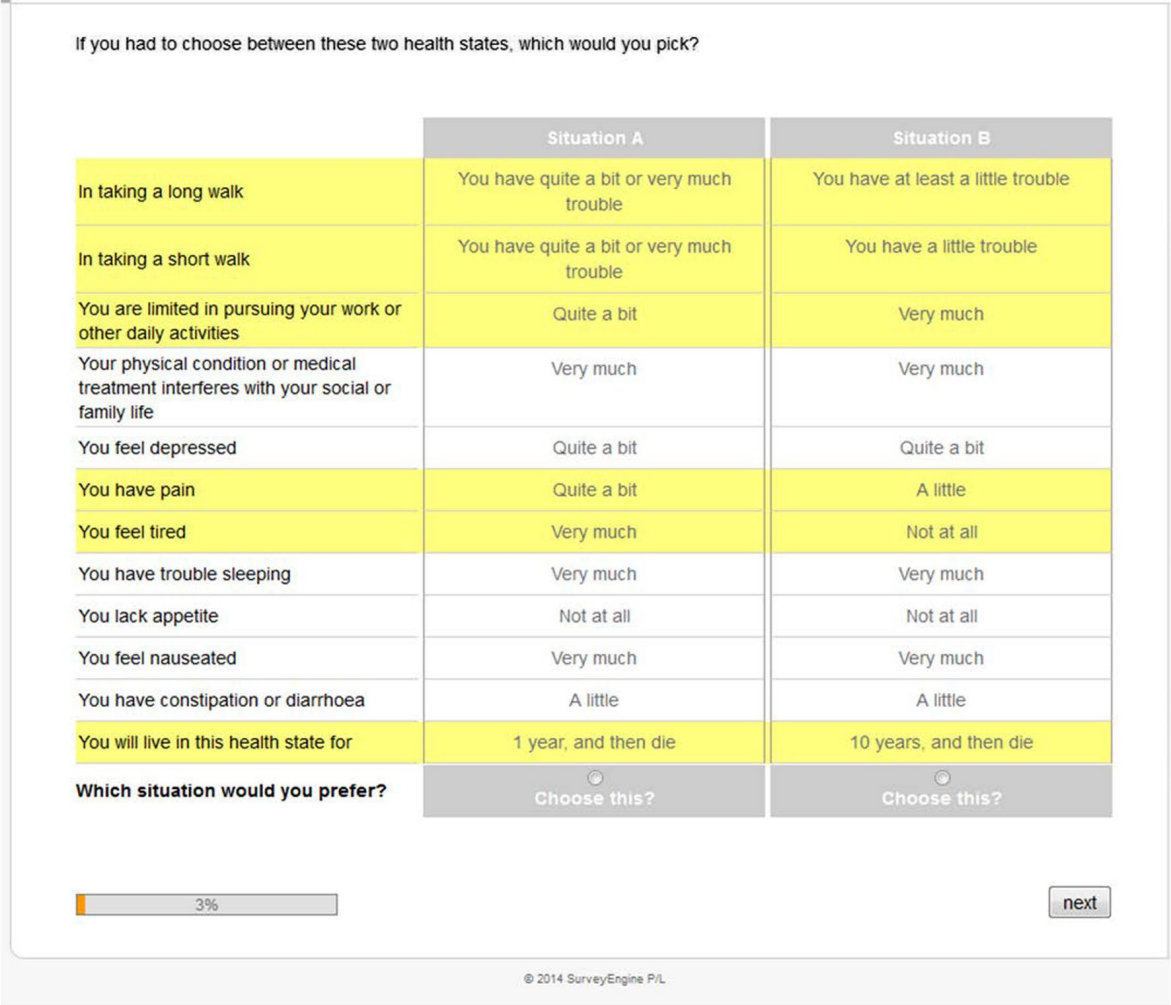
Example DCE choice set form QLU-C10D valuation survey

For QLU-C10D valuations a standardised methodology is in place. The survey comprises questions on sociodemographic information, 16 DCE choice sets (selected out of a total of 960; described below), self-report questionnaires on health status (QLQ-C30, Kessler-10, EQ-5D-3L), and feedback questions on the clarity of health state presentations (assessed on a 5-point Likert scale from ‘very clear’ to ‘very unclear’), the difficulty in comparison to other surveys (respondents are asked to compare to any other survey they might have participated in which could be none in the case of cancer patients or could be questionnaire studies they might be familiar with; response options are ‘easier’, ‘similar’, ‘more difficult’, and ‘can’t tell’;), the difficulty to make a decision between the health states (assessed on a 5-point Likert scale including the options ‘very difficult’, ‘difficult’, ‘neither/nor’, ‘easy’, ‘very easy’), and the strategy on how a decision was reached (options: ‘no strategy’, ‘focus on a few aspects’, ‘focus on highlighted aspects’, ‘focus on most aspects’, ‘focus on all aspects’, and ‘other strategy’). All survey material is provided in the supplementary material. The survey is administered web-based. Each DCE choice set comprises two hypothetical QLU-C10D health profiles (i.e., the 10 HRQOL domains with different levels of impairments on 4 levels from “not at all” to “severe”) and survival times in that health states (one, two, five, or ten years). To keep cognitive burden on a manageable level, impairments on only five domains differed between the two options in each choice set (highlighted in yellow). The respondents are asked to select their preferred health profile (see example in Fig. 1). More details on the DCE and the valuation survey can be found in prior publications [12–14, 16]. The QLU-C10D valuation methodology has been intensively investigated, including testing the impact of different graphical presentations, impact of ordering of attributes, and test–retest reliability [13–15].

### Mixed-methods approach for pilot testing in cancer patients

Based on Collins [23], Mullin et al. [24] and Atkinson et al. [25] the following aspects were assessed with regard to the applicability of the QLU-C10D valuation methodology in cancer patients: comprehension, i.e. understanding of the task (e.g. How clear/unclear is the purpose of the survey/this explanation to you? Could you repeat this in your own words?), retrieval, i.e. the information processing strategy including recall of information (e.g. Do you have a particular strategy?), judgement, i.e. the process of formulating an answer to each question (e.g. How easy/difficult was your choice?), response (e.g. How do you feel about your choice?), and burden, i.e. the perceived importance or reasonability of the task which is linked to collaboration motivation (e.g. How relevant do you consider this tasks to be? Would you consider the tasks suitable for other patients as well? What would you change?).

This was achieved by employing a mixed-methods approach.

The qualitative part comprised a cognitive interview with verbal probing [26] with cancer patients covering the mental process in capturing the provided information and in giving responses. The interview was performed alongside the completion of the QLU-C10D valuation survey. The quantitative part encompassed the comparison of responses to the feedback questions incorporated in the survey between patients and matched control group respondents. Survey material has been provided in the supplementary files (Additional file 1: Appendix A).

### Analyses and sample size considerations

Sample size considerations were based on the recommendations of Lancaster et al. [27], and specifically on Morse [28] and Glaser and Strauss [29] focussing on the concept of content saturation for qualitative approaches. The concept of content saturation with approx. 30 participants was again confirmed in a content analysis involving 560 studies by Mason et al. [30].

Qualitative data was analysed based on the Grounded Theory Approach, described by Glaser [31]. Interview data were independently reviewed by two researchers performing inductive coding using Microsoft Excel.

Quantitative data was analysed using Chi-square tests for comparing frequencies (feedback questions). To show that included patients and general population controls indeed differed with regard to functioning and health status we also compared EORTC QLQ-C30 scores (assessed as part of the valuation survey—see above). This was done using Mann–Witney-U tests. A significance level of 0.05 was applied. Statistical analyses were conducted using IBM SPSS 23.0.

## Results

### Sample characteristics

We included a total of 48 cancer patients (mean age 59.9 years, SD 13.5; 46% female). Diagnoses were mixed (breast, haematological, lung, neuroendocrine, thyroid, gastrointestinal, colorectal, and other) and all but four patients were under active therapy (radiotherapy, chemotherapy, or nuclear therapy). Sample characteristics are shown in Table 1. We matched 192 respondents from the general population sample according to age, sex, and education. Hence, regarding sociodemographic parameters, participants only differed significantly regarding marital status, with more patients being single and more participants of the control group being divorced (*p* < 0.001).

**Table 1:** Sample characteristics

	Patient sample (N = 48)		Control group (N = 192)	
Mean age (SD)	59.9 (13.5)		59.0 (12.1)	
	N	%	N	%
Sex				
Female	22	45.8	88	45.8
Male	26	54.2	104	54.2
Marital status				
Single	12	25.0	18	9.4
Married/partnership	29	60.4	120	62.5
Separated/divorced	4	8.3	44	22.9
Widowed	3	6.3	10	5.2
Highest education				
Elementary school	1	2.1	4	2.1
Secondary lower/apprenticeship	29	60.4	116	60.4
A-level	10	20.8	40	20.8
University level	8	16.7	32	16.7
Cancer diagnosis				
Breast	4	8.3		
Haematooncological	16	33.3		
Lung	4	8.3		
Neuroendocrine	5	10.4		
Thyroid	10	20.8		
Gastrointestinal/colorectal	3	6.3		
Other	6	12.6		
Current therapy				
Chemotherapy	24	50.0		
Radiotherapy	9	18.8		
Nuclear therapy	11	22.9		
Surgery only	4	8.3		

The sample characteristics of the patient sample and the general population control group from an internet panel. Patients differed significantly from the control group regarding the marital status (*p* < 0.001)

SD, standard deviation

**Fig. 2:**
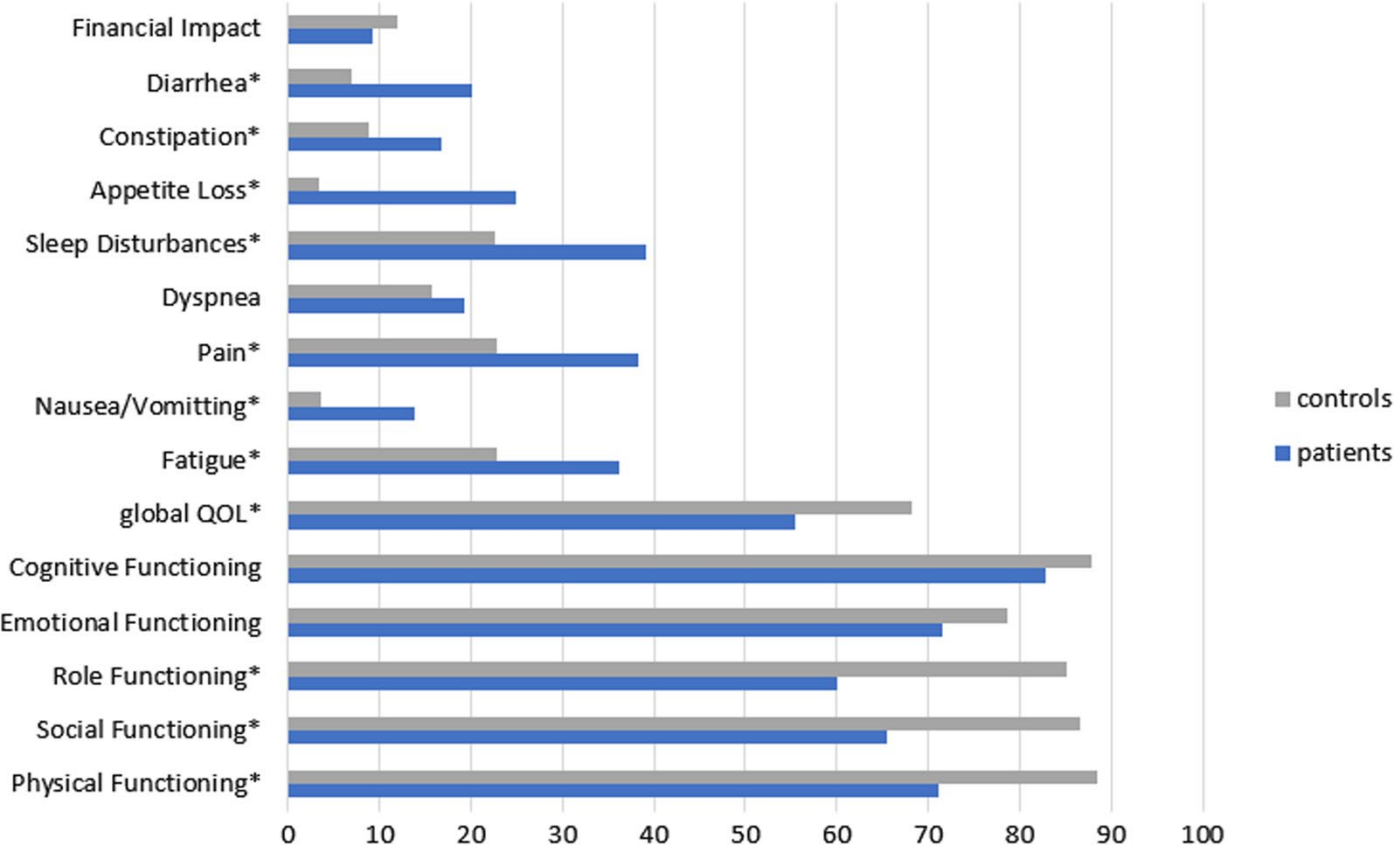
Health-related quality of life differences between patients and general population controls as measured with the QLQ-C30. The group differences in HRQOL domains regarding the cancer patient sample (N = 40) and the control group from the general population (N = 192). Except from the scale for constipation (*p* = 0.05), all significant differences showed *p* values ≤ 0.003; all statistical significant differences met the criteria for a clinical relevance based on Cocks et al. [31]

Patients and general population controls differed with regard to functioning and health status measured by the EORTC QLQ-C30. Compared to the general population controls, patients’ HRQOL was significantly worse on 11 of 15 domains of the EORTC QLQ-C30 (see Fig. 2). Differences that were statistically significant met the criteria for clinical relevance according to Cocks et al. [32]. We identified large differences (> 19 points) regarding social functioning, and medium differences (> 8 points) regarding physical and role functioning, as well as regarding fatigue, nausea/vomiting, pain, sleep disturbances, appetite loss, and diarrhoea. Global quality of life of the cancer patients differed also on a medium level (approx. 13 points) from the general population controls. As EORTC QLQ-c30 data was missing for eight patients, we conducted a sensitivity analysis by imputing normative data from the Austrian general population [33]. Results did not change.

### Results from cognitive interviewing

Overall comprehension of the DCE survey was good with 90% of the patients finding the task clarity to be good. The retrieval processes for the decision process included the subjective relevance of the attributes (i.e. decision strategy) in the DCE (i.e. HRQOL domains and survival time); 46% stated that the survival time was the most important attribute and 38% made their decision based on HRQOL impairments, with pain being most often explicitly named.

**Fig. 3:**
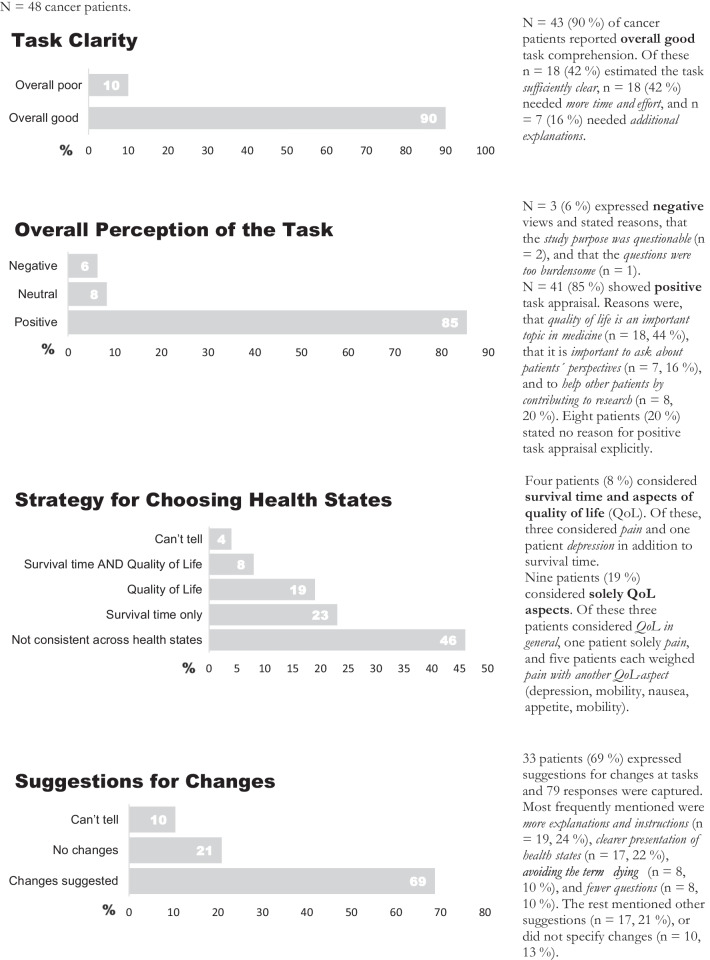
Results of the cognitive debriefing interviews. N = 48 cancer patients

Overall, patients considered the task to be positive (85%), mainly because they considered quality of life research to be an important topic in medicine (38%). The most frequent suggestions for survey improvement were providing a clearer presentation of the different health states (17%) and providing additional explanation and instructions (19%). Potential inappropriateness for some patient groups was mentioned by 4 patients only (8%), who suggested to avoid the term “dying” in the survey in order to make it suitable for other patients. Detailed results of the cognitive interviews are presented in Fig. 3. Results on patient utilities cannot be reported from this small number of respondents, but required finalizing the field study.

### Results from quantitative comparision

**Fig. 4:**
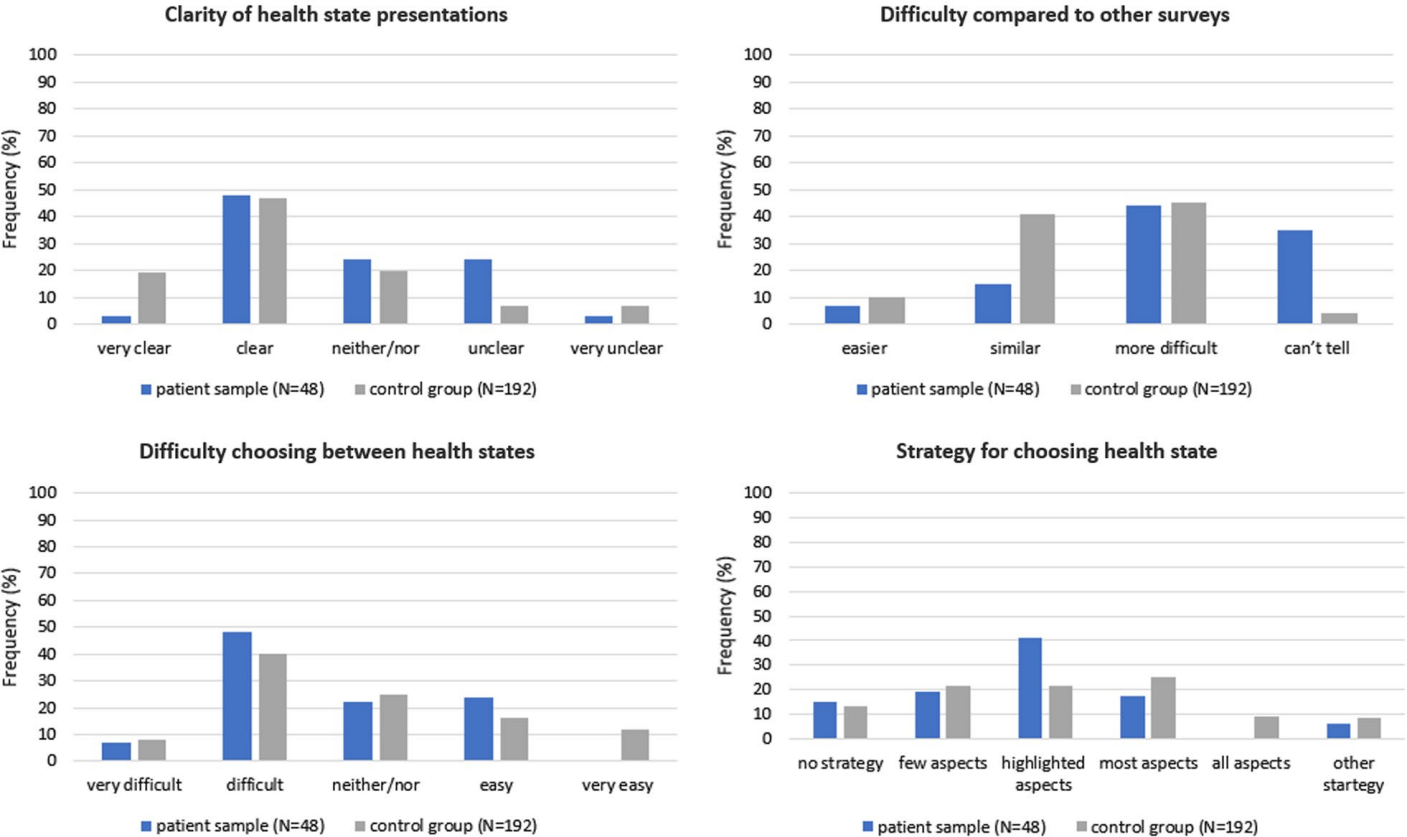
Results from the QLU-C10D valuation survey feedback questions. The results from the feedback questions of the QLU-C10D valuation survey. Whereas differences between cancer patients (N = 48) and the control group (N = 192) regarding the presentation clarity (i.e. comprehension) and difficulty compared to other surveys (i.e. judgement and response) was significant (*p* = 0.003 and *p* < 0.001), there were no significant differences in the perceived difficulty when choosing between two health states (*p* = 0.344), nor regarding the strategy for choosing a health state (*p* = 0.140)

Regarding comprehension of the DCE survey, patients found the presentation of the health states to be less clear than the general population sample (*p* = 0.008). Fewer patients rated the tasks as “very clear” while about the same percentage of patients and controls rated the tasks as “clear”. The percentage of patients rating the tasks as “unclear” with 24% was clearly higher than in the controls (7%) Patients furthermore differed from the general population with regard to their ratings on the survey’s difficulty in comparison to other survey (*p* < 0.001). This difference was mainly due to a much higher percentages of patient who used the “can’t tell” category. There was no difference between the groups concerning how difficult it was to choose between the health states in each choice set (*p* = 0.344). Likewise, no statistically significant difference with regard to stated strategy for choosing between health states was found (*p* = 0.104). Details can be seen in Fig. 4.

## Discussion

Our study results approve the QLU-C10D valuation survey for use in cancer patients due to the following reasons based on Collins [23], Mullin et al. [24] and Atkinson et al. [25]: Sufficient comprehension can be considered based on clarity ratings gathered from qualitative interviews and quantities comparisons with controls the following aspects were assessed with regard to the applicability of the QLU-C10D valuation methodology in cancer patients: comprehension, i.e. understanding of the task (e.g. How clear/unclear is the purpose of the survey/this explanation to you? Could you repeat this in your own words?), retrieval, i.e. the information processing strategy including recall of information (e.g. Do you have a particular strategy?), judgement, i.e. the process of formulating an answer to each question (e.g. How easy/difficult was your choice?), response (e.g. How do you feel about your choice?), and burden, i.e. the perceived importance or reasonability of the task which is linked to collaboration motivation (e.g. How relevant do you consider this tasks to be? Would you consider the tasks suitable for other patients as well? What would you change?).

Results form qualitative and quantitative approaches considered together, it has been shown that the imposed burden, despite including personal trade-offs between HRQOL and survival time, was perceived manageable by patients and that although patients were in a compromised health state, which may add to cognitive burden, the survey was in general acceptable for them and they were able to manage the rather complex DCE tasks involving 10 health domains and an expected survival time. In overall difficulty was not considered to be an issue of concern by the patients in the interviews. Patients did not comment on the time frames of survival in the hypothetical scenarios, and few were bothered by the term “dying” in the tasks and would have suggested to avoid this before giving the survey to other patients. Lack of clarity of information/explanation was identified by some patients.

Patients’ feedback differed from that of the general population controls in very few aspects. A high percentage of patients could not make a comparison between the difficulty of the present survey and other surveys. This is not surprising as internet panel members are much more survey-trained and participants recruited via Survey Engine usually are even familiar with DCEs. For the same reason a higher percentage of patients may have rated task clarity lower than controls did. As overall clarity ratings were very highand patients in the interview stated that additional effort, time and explanation made the tasks easier to understand clarity does not seem to be an issue of concern but will need to be addressed in the presentation of the survey to patients.

Most importantly though, there was no difference in overall perceived task difficulty between patients and general population respondents. In overall, task difficulty ratings did not raise severe concerns and appeared neither too difficult nor too easy. Our considerations with regard to difficulty were that task that are “too easy” might comprise some sort of dominant choices, i.e. health situations which do not require the respondent to make a trade-off (e.g. a situation with good QOL and longer survival vs a situation with poor QOL and shorter survival) whereas tasks that are too hard might result in fatigued respondents who make guesses and mistakes. The numbers we found compare well to the results for the general population [14] and are within the range of acceptable difficulty level reported in the literature, which is, though a bit scarce. The lack of qualitative research in DCE research in general has been pointed out in a systematic review by Vass et al. [34]. We identified a study by Mulhern et al. [35] comparing a DCE with a time-trade off (TTO) for EQ-5D-5L health states that showed that 57% of the respondents considered the DCE tasks difficult to answer and 63% considering the TTO tasks difficult to answer. Norman et al. [36] identified a clearly lower number of 11% of respondents to rate tasks of a DCE for EQ-5D-5L health states to be either difficult or very difficult. Other examples in the context of health are Skedgel et al. [37] who investigated societal preferences for the allocation of health care resources and found 65% to rate the presented DCE questions somewhat or extremely difficult to answer, and Green and Gerard [38] with a similar objective where as many as 68% considered it fairly difficult or very difficult to complete the DCE tasks.

It is a limitation of our study that cannot provide information from qualitative interviews from the general population sample as well for a direct comparison. This would shed additional light on potential differences between patient and general population perceptions of the DCE tasks and the type of study in general. A further limitation is that we draw on a convenience sample of patients and it was not possible to include patients in a very compromised health state. The interview was lengthy and may have posed a questionable high burden on these patients. Their perspective on the issues in questions though will be important though in further research on patient valuations and may require a different approach of obtaining it.

In order to further reduce the burden for patients while maintaining comparability of the survey with the general population, adaptations for the field study may include improvements of information and explanation while keeping all the survey elements as they are. This can be done by setting up a website and a telephone hotline to provide additional information and explanation to patients and to address potential emotional burden.

To date, there is no consensus if there is actually a systematic difference in health valuations depending on who is the source of information (general population or patients), two meta-analyses reporting conflicting results [39, 40]. Yet, potential consequences for health economic evaluations need to be considered and further explored. An important argument to investigate patient preferences for disease-specific PBMs is that respondents from the general population usually can relate to generic HRQOL issues, such as pain, but may have more difficulty imagining and hence valuing the impact of severe fatigue, for example, on a purely hypothetical basis.

## Conclusion

Patient valuations for the QLU-C10D will contribute to the ongoing discussion on the need of rethinking which population is more relevant for providing health preferences to estimate utilities [41–43]. The results presented here add to the scarce literature on patient valuations and provide a positive outlook with regard to feasibility and acceptance in this specific target group.

## Supplementary Information

**Additional file 1.** Appendix A.

## Data Availability

The datasets generated and/or analysed during the current study can be found in the supplemental material.

## Abbreviations

CUA: Cost-utility analyses
DCE: Discrete choice experiment
EORTC: European Organisation for Research and Treatment of Cancer
QLU-C10D: Quality of life utility core 10 dimensions
HRQOL: Health-related quality of life
PBM: Preference-based measure
QALY: Quality adjusted life year
QLQ-C30: Quality of Life Questionnaire Core 30
SD: Standard deviation
