# Supplementary material for: The EORTC QLU-C10D discrete choice experiment for cancer patients: a first step towards patient utility weights
Source: J Patient Rep Outcomes. 2022 May 4;6:42. doi: 10.1186/s41687-022-00430-5 (PMC9068836; doi:10.1186/s41687-022-00430-5)
Supplement: Supplementary file 1 — Additional file 1. Appendix A. [file 41687_2022_430_MOESM1_ESM.pdf]

QLU-C10D user manual

Authors: Eva Gamper, Femke Jansen, Matthias Buettner, Madeleine King, Richard Norman, Georg Kemmler, Virginie Nerich, Bernhard Holzner, Micha Pilz, Simone Seyringer, Andrew Bottomley

0. Aim of user manual and target audience

This user manual addresses researchers and industry members who want to collect cancer-specific health-related quality of life (HRQOL) data and use that data for the calculation of preference-based scores and quality-adjusted life years (QALYs). It provides information and guidance on the proper legal and scientific use of the EORTC QLU-C10D including modes of administration, scoring, and results reporting and translations. It also provides guidance on the development of new value sets. Acknowledging the various professional backgrounds of potential users, part 1 of the manual provides some theoretical background on preference-based and non-preference based HRQOL research and an overview of different fields of application. Part 2 provides guidance on the practical administration of the EORTC QLU-C10D. The manual may be used in conjunction with other EORTC manuals and the EORTC QLG website (https://qol.eortc.org/) which contains information on most current developments. Weblinks to respective resources are provided. For further information and assistance regarding the QLU-C10D, such as licencing, translations and ongoing investigations, you can also contact the EORTC Quality of Life Department (QLD) directly (*contact information here*).

TABLE OF CONTENTS

PART 1 - BACKGROUND INFORMATION 3

Key message box 3

1. RESEARCH GROUPS AND INSTITUTIONS 3

1.1. The EORTC Quality of Life Group (QLG) 3

1.2. The EORTC Quality of Life Department (QLD) 4

1.3. The MauCa Consortium 4

2. THEORETICAL BACKGROUND AND TERMINOLOGY 4

2.1. Measuring and valuing health – HRQOL profiles versus preference-based measurement 4

2.2. Generic and disease-specific preference-based measures 6

2.3. The EORTC QLG measurement system 7

2.4. What shall I use: an EORTC HRQOL profile measure or the EORTC QLU-C10D? 8
PART 2: EORTC QLU-C10D – GUIDELINES FOR USERS 9
Terminology overview 9
3. The EORTC QLU-C10D 9
3.1. The development of the QLU-C10D out of the QLQ-C30 9
3.3. EORTC QLU-C10D valuations 13
4. PROPERTIES AND APPLICATION 14
4.1. PSYCHOMETRIC PROPERTIES 14
4.2. ADMINISTRATION 14
4.3. SCORING AND CONVERTING HEALTH STATES TO AN INDEX VALUE .. 15
4.4. ORGANISING QLU-C10D DATA 16
4.5. QLU-C10D scoring example 17
5. PRESENTATION OF RESULTS 20
5.1. QLU-C10D index score 20
5.2. Health profiles 20
6. AVAILABLE TRANSLATIONS AND FORMATS 20
7. QLU-C10D NORMATIVE DATA 22
8. CAN I CONVERT UTILITIES OBTAINED WITH ANOTHER INSTRUMENT INTO QLU-C10D UTILITIES? 23
9. HOW TO OBTAIN THE QLU-C10D AND TERMS OF USE 23
10. HOW TO REFER TO THE QLU-C10D IN PUBLICATIONS 23
11. WHERE TO FIND INFORMATION ABOUT CURRENTLY ONGOING EORTC QLU-C10 STUDIES 23
12. HOW TO DEVELOP ADDITIONAL EORTC QLU-C10D VALUE SETS 23
References 24
Appendix A 27
QLU-C10D scoring algorithm for POLAND 27
QLU-C10D scoring algorithm for the NETHERLANDS 29

PART 1 - BACKGROUND INFORMATION

Key message box

- Research groups and institutions: EORTC QLG and QLD, MauCa consortium
- Health-related quality of life (HROQL):
  - a multidimensional concept that encompasses a patient's subjective perception of the effect of disease and treatment on physical, psychological and social aspects of daily life
  - assessed using patient-reported outcome measures (PROMs)
- There are two large groups of PROMs:
  1. HRQOL profile measures:
     - result in individual scores for different HRQOL dimensions (e.g. separately for physical functioning, psychological, pain, etc.)
     - scores represent the extent of the patient's well-being or the severity of symptoms
     - designed for use in clinical studies and clinical routine
  2. Preference-based measures (PBMs)
     - result in one single score that represents the "value" of the reported health state
     - values are derived from health preferences that have been provided by a target group (usually the general population)
     - designed for use in economic evaluations, often to calculate quality-adjusted life years (QALYs)

1.1. The EORTC Quality of Life Group (QLG)

The European Organisation for Research and Treatment of Cancer (EORTC) Quality of Life Group (QLG) is a multiprofessional, international group of researchers and clinicians dealing with cancer. It formed in 1980 and has dedicated its activities to health-related quality of life (HRQOL) in oncology ever since. This encompasses advocating HRQOL measurement in clinical studies and practice by providing a refined HRQOL measurement system, driving forward methodological research in the field, and, in cooperation with the EORTC Quality of Life Department (QLD), supporting EORTC disease-specific groups on the incorporation of HRQOL outcomes in EORTC clinical trials. It is open to researchers and clinicians all over the world who have an interest in HRQOL research in oncology. The EORTC being a registered not-for-profit organisation means that all income is used for research, education and user support.

The EORTC QLG website (https://qol.eortc.org/) provides detailed information and updates on the EORTC HRQOL measurement system, including user manuals, available language versions key references, licencing information, upcoming events and ongoing research projects.

1.2. The EORTC Quality of Life Department (QLD)

The Quality of Life Department (QLD) is a division of the EORTC Data Center dedicated to a coherent policy and providing a standard approach in conducting HRQOL research in cancer clinical trials. The principal tasks of the QLD are to support the incorporation of HRQOL data collection into clinical trial protocols, to establish an adequate infrastructure for the data management of studies, which include HRQOL endpoints, and to guide analysis of HRQOL data in EORTC clinical trials. It also manages the translation and licensing of EORTC HRQOL measures.

1.3. The MauCa Consortium

The Multi-attribute Utility in Cancer (MauCa) consortium is an international group of HRQOL researchers who developed the EORTC QLU-C10D health state description system and valuation methodology.

2. THEORETICAL BACKGROUND AND TERMINOLOGY

2.1. Measuring and valuing health – HRQOL profiles versus preference-based measurement

HRQOL is a multidimensional concept and encompasses a patient's subjective perception of the effect of their disease and treatment on physical, psychological and social aspects of daily life (Bottomley, Pe et al. 2016). Several patient-reported outcome (PRO) instruments exist that can be used to measure a patient's HRQOL. PRO instruments can broadly be divided into two groups, namely the HRQOL profile measures and preference-based measures (PBMs) (Drummond 2005). The two types of instruments represent two different approaches to HRQOL assessment (measuring versus valuing health) and their results serve different research purposes (clinical research versus health economics research).

2.1.1. HROQL profiles

HRQOL profile measures are questionnaires which result in a number of individual domain scores constituting a self-reported HRQOL profile. In general, they include aspects of functioning (e.g. physical functioning) and, depending on their target group, generic (e.g. pain) or disease-specific symptoms. They are used predominantly in clinical research, e.g. as measures of secondary outcomes in trials or in observational studies and registries to investigate HRQOL trajectories and to evaluate the impact of disease and treatment on different aspects of functioning and well-being. HRQOL profile measures increasingly find their way into clinical practice where they serve as HRQOL screening tools and allow for early detection of symptoms as well as improve clinical care. Commonly used HRQOL profile measures in the field of cancer include the EORTC Quality of Life Questionnaire Core 30 (EORTC QLQ-C30) and the Functional Assessment of Cancer Therapy – General (FaCT-G).

2.1.2. Preference-based measures (PBMs)

PBMs are designed to take into account the preference towards a particular health profile or outcome (Drummond 2005), i.e. to "value" health. They consist of a so-called health classification system, i.e. a set of health/HRQOL aspects, which may take on different severity levels, and a preference-based scoring algorithm, i.e. similar to HRQOL profile measure include a number of questions on different HRQOL aspects and a certain number of response options. Their scoring is quite different from that of a profile measure. The outcome of a PBM is a single number, also known as a "utility" that expresses the "value" which has been assigned to the described health state by the target population. The utility score in theory ranges from 0 (representing being dead, or a state equivalent to it) to 1 (representing optimal/best imaginable health). In practice, depending on the statistical model, scores below 0 (i.e. health states worse than death) are possible. PBMs are also called "utility instruments". The values assigned to the health states described by the PBM have to be elicited previously in the target population (mostly the general population) in a "valuation study" (usually separately for countries) and then form a so-called "value set".

PBMs are usually used in cost-utility analyses (CUAs). CUA is a method in economic evaluations to compare different health interventions with regard to their costs and consequences on HRQOL and survival. A CUA's final outcome is the incremental cost-effectiveness ratio (ICER), also more precisely often called incremental cost-utility ratio (ICUR), which is the difference in costs between two interventions divided by their difference in effect. The treatment effect hereby can be expressed in quality-adjusted life years (QALYs), which is the survival time adjusted for quality of life, i.e. for the utility, which can be assessed by a PBM (Drummond 2005).

Figure 1 shows a brief overview of the characteristics of HRQOL profile measures and PBMs.

HRQoL PROFILE *VERSUS* PREFERENCE-BASED MEASURES

HRQoL PROFILE MEASURE	PREFERENCE-BASED MEASURE
Psychometric approach	Utility approach
A respondent's describes his/her own HRQoL	Scores represent the value of health states
Domains of HRQoL are kept separate, a series of domain scores describe a HRQoL *profile*	Domains of HRQoL are combined into a single *index*
Each domain score is typically derived from several items (questions)	Typically one item (question) per domain
Typically items (questions) are unweighted	Domains are weighted
Ordinal scales	Weights are derived using a preference-based method
No anchors: minimum and maximum values are a function of scoring algorithm	Interval scale, anchored at 0 (death) and 1 (full health), values worse than death are possible
Typically average or sum of items	
Often linearly transformed to 0-100 range	

Figure 1: Overview characteristics of HRQOL profile measures versus preference-based measures

2.2. Generic and disease-specific preference-based measures

There are two overall types of PBMs to be distinguished: generic and disease-specific.
Generic PBMs are currently predominantly used for the assessment of utilities for CUAs. They use broad, basic outcome dimensions, which make them applicable for most medical conditions, thereby allowing comparability between different diseases and treatments (Dolan 1997, Drummond 2005, Rowen, Zouraq et al. 2017). The most prominent generic utility measures are the EQ-5D-3L (The EuroQol Group 1990), the EQ-5D-5L (Herdman, Gudex et al. 2011), the Short-Form Six-Dimensions (SF-6D) (Brazier, Roberts et al. 2002), the Health Utility Index Mark 2 (HUI2) (Horsman, Furlong et al. 2003), the Health Utility Index Mark 3 (HIU3) (Grootendorst, Feeny et al. 2000), and the 15D (Sintonen 2001). Even though generic PBMs are the preferred choice for performing CUAs in a health economic setting, various aspects have been criticised, especially regarding their appropriateness in certain settings, such as in oncology. Their measurement properties vary across conditions as some arguably lack sensitivity in specific health states (Bharmal and Thomas 2006, Rowen, Young et al. 2012, Rowen, Brazier et al. 2017) (Rowen, Young et al. 2012). This is potentially a result of a low number of dimensions or response categories. Alternatively, it may result from the included dimensions not measuring the issues relevant to the target patient population. Independent of the potential explanations, inappropriate scale coverage in specific conditions, mostly ceiling effects, are an issue in generic PBMs (Brazier, Roberts et al. 2004, Sullivan, Lawrence et al.

2005, Janssen, Pickard et al. 2013, Conner-Spady, Marshall et al. 2015). This means that some of these instruments report full health (i.e. a utility of 1) for the patient even though s/he has an impaired quality of life or a reduction in health status (Pickard, Ray et al. 2012, van Dongen-Leunis, Redekop et al. 2016).

Therefore, there is an ongoing debate on the potential added value of disease-specific PBMs which might be able to overcome many of these weaknesses.

Disease-specific PBMs may overcome many of the limitations of generic PBMs. They have been shown to be able to detect small changes and to discriminate better between health states of patients (Rowen, Brazier et al. 2017). Some show a better validity and responsiveness in the disease under investigation (Wiebe, Guyatt et al. 2003, Krahn, Bremner et al. 2007). However, on the downside, disease-specific PBMs are suspected of exaggerating health effects due to possible focusing effects of the instrument (Brazier and Tsuchiya 2010, Versteegh, Leunis et al. 2012). A further major concern regarding disease-specific PBMs is that they need to be developed and evaluated for each specific disease. Since it is known that different PBM may result in different utility weights (Pickard, Ray et al. 2012, Färkkilä, Torvinen et al. 2014), this also raises questions on how disease-specific instruments can be reliably compared with the results from generic instruments in health economics evaluations.

Hence, while the psychometric approach seems to be evident, i.e. the need to evaluate the potential gain in accuracy and appropriateness by developing and comparing novel disease-specific PBMs with traditional generic ones, how a variety of measures in regulatory practice can be worked with needs clarifying.

2.3. Generic and disease-specific preference-based measures

2.4. The EORTC QLG measurement system

A core activity of the EORTC QLG is the continuous extension and refinement of the EORTC HRQOL measurement system. This consists of a range of HRQOL profile measures and one PBM (the QLU-C10D). The following provides a brief overview – for details please refer to https://qol.eortc.org/quality-of-life-group/#.

A main body of the EORTC measurement system is the *Quality of Life Questionnaire-Core 30* (QLQ-C30) as the core instrument (and its computer-adaptive version, the *EORTC CAT)* which can be complemented with site- and treatment-specific modules, all covering functional and symptomatic aspects of the (specific) disease and/or treatment. In addition stand-alone measures are available for specific purposes, such as for the assessment of patient satisfaction or communication.

Furthermore, the EORTC provides two kinds of item collections which allow the construction of custom-built question sets, an item response theory (IRT-) calibrated item bank, which

comprises all items from the EORTC CAT (i.e. covering the content of the domains of the QLQ-C30) and an item library, a simple collection of all questions used in EORTC instruments. For details on the use of the item bank and item library please refer to the QLD *(contact information here)*.

These EORTC instruments produce HRQOL profiles and were designed for the use of outcome measurement in cancer clinical studies and HRQOL screening in clinical practice.

The development of the Quality of Life Utility-10 Dimensions (QLU-C10D) allows data collected with the EORTC QLQ-C30 to now also be used in health economics research by providing a preference-based scoring algorithm for the calculation of cancer-specific health utilities.

2.5. What shall I use – an EORTC HRQOL profile measure or the EORTC QLU-C10D?

A health profile measure results in an array of scores representing individual dimensions/domains of HRQOL. This is of interest where a differential measurement of effects of a disease/health condition or health intervention on single or various HRQOL domains is required. This may be the case in clinical studies where interventions target a specific aspect of HRQOL as well as in clinical practice where tracking a set of symptoms and functioning aspects supports disease management, or in disease registries where the aim may be to learn about trajectories covering various aspects of HRQOL. Some profile measures also provide the possibility of calculating a summary score across different HRQOL domains for research objectives including a more general HRQOL endpoint.

PBMs result in a single number (index score), which usually represents a continuum from optimal/best imaginable health (1) to death (0) and consists of the combination of HRQOL domains as a description of overall health. Some PBM algorithms allow values below 0 representing health states considered worse than death. The index score incorporates both, a respondent’s subjective health status at a certain point of time and the preference value/utility obtained from a defined target group (e.g. general population, patients, health care professionals) for that overall health status. Such an outcome is predominantly interesting in (pharmaco-) economic research, mostly in cost-utility-analyses, where it serves as a correction factor for survival time, i.e. for the calculation of quality-adjusted life years (QALYs) (see section 2.1.2). The respondent’s health state descriptions (i.e. before applying the preference-based scoring algorithm is performed) may be presented as health profiles. However, these are usually based on single items which come with a larger measurement error than scales based on several items.

Most profile measures do not allow utility calculation and, vice versa, most preference-based measures are not designed for a sound analysis of health profiles. Therefore, appropriateness of the respective measure’s psychometric properties needs to be evaluated before the background of the primary study purpose. This includes assessing whether it has been validated for the specific target group such as the specific disease and county/language…

PART 2: EORTC QLU-C10D –GUIDELINES FOR USERS

Terminology overview

Preference-based measure (PBM):

- a HRQOL instrument that results in a single index value expressing the “value” of a certain health state to be used in health economics research (see section 2.1.2)
- synonyms: utility instrument, multi-attribute utility instrument (MAUI)

Utility:

- the “value” assigned to a health state by a target population (mostly the general population of a country) by providing preferences for health states
- anchored at 0 (representing death) and 1 (representing best imaginable health) (see 2.1.2)
- synonyms: health state utility value (HSUV), utility value

Value set: the utility values for the health states described by a PBM provided by a specific population (usually the general population of a country)

3. The EORTC QLU-C10D

3.1. The development of the QLU-C10D out of the QLQ-C30

The EORTC Quality of Life Utility-Core 10 Dimensions (QLQ-C10D) is a preference-based measure based on the profile HRQOL instrument of the EORTC, the QLQ-C30 (Aaronson et al 1993). Its health state description system was developed by the Multi-Attribute Utility Cancer (MAUCa)-Consortium and included a thorough mixed methods approach to domain selection and evaluation based on an item-response theory analyses of a large international QLQ-C30 data set and qualitative interviews with cancer patients, health care experts, and HRQOL researchers (King et al. 2016).

The rationale for its development was the increasing knowledge on measurement issues of generic PBMs in certain populations and for specific purposes and the lack of cancer-specific PBMs which may better capture cancer-specific HRQOL aspects. Also generic PBMs are predominantly used in health economics investigations. In contrast to cancer-specific HRQOL measures they are not routinely included in cancer clinical trials as from a profile measure perspective they do not provide a lot of additional information.

3.2. What is a QLU-C10D health state?

The QLU-C10D incorporates a health description system consisting of 13 items of the parent instrument QLQ-C30. The items form 10 domains (physical functioning (PF), role functioning (RF), social functioning (SF), emotional functioning (EF), pain (Pa), fatigue (Fa), sleep (Sl), appetite (Ap), nausea (Na), and bowel problems (Bo)) (see Figure 2).

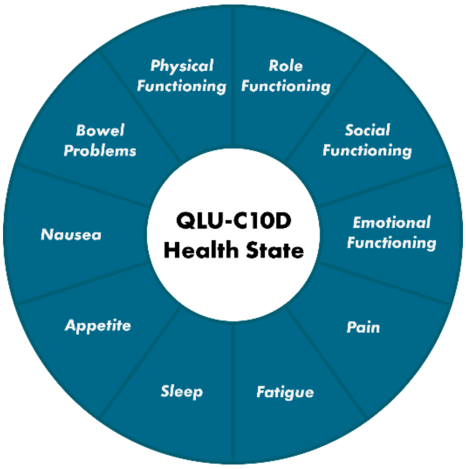

Figure 2: QLU-C10D Health State

Each domain can take on four levels which describe levels of impairment:
Level 1: no problems
Level 2: some problems
Level 3: quite a few problems
Level 4: severe problems

A QLU-C10D health state is described by a 10-digit code providing the level of impairment per domain, i.e. a total of 4^{10}=1048576 health states can be described with the best being 1111111111 (no problems in all domains) and the worst 4444444444 (severe problems in all domains).

Table 1 shows the QLU-C10D health state description system including translation from QLQ-C30 response levels to QLU-C10D domain levels. The recall period is the same as for the QLQ-C30 items.

EORTC QLQ-C30 (version 3)

We are interested in some things about you and your health. Please answer all of the questions yourself by circling the number that best applies to you. There are no "right" or "wrong" answers. The information that you provide will remain strictly confidential.

Please fill in your initials:
Your birthdate (Day, Month, Year):
Today's date (Day, Month, Year): 31

	Not at All	A Little	Quite a Bit	Very Much
1. Do you have any trouble doing strenuous activities, like carrying a heavy shopping bag or a suitcase?	1	2	3	4
2. Do you have any trouble taking a long walk?	1	2	3	4
3. Do you have any trouble taking a short walk outside of the house?	1	2	3	4
4. Do you need to stay in bed or a chair during the day?	1	2	3	4
5. Do you need help with eating, dressing, washing yourself or using the toilet?	1	2	3	4

**During the past week:**	Not at All	A Little	Quite a Bit	Very Much
6. Were you limited in doing either your work or other daily activities?	1	2	3	4
7. Were you limited in pursuing your hobbies or other leisure time activities?	1	2	3	4
8. Were you short of breath?	1	2	3	4
9. Have you had pain?	1	2	3	4
10. Did you need to rest?	1	2	3	4
11. Have you had trouble sleeping?	1	2	3	4
12. Have you felt weak?	1	2	3	4
13. Have you lacked appetite?	1	2	3	4
14. Have you felt nauseated?	1	2	3	4
15. Have you vomited?	1	2	3	4
16. Have you been constipated?	1	2	3	4

Please go on to the next page

**During the past week:**	Not at All	A Little	Quite a Bit	Very Much
17. Have you had diarrhea?	1	2	3	4
18. Were you tired?	1	2	3	4
19. Did pain interfere with your daily activities?	1	2	3	4
20. Have you had difficulty in concentrating on things, like reading a newspaper or watching television?	1	2	3	4
21. Did you feel tense?	1	2	3	4
22. Did you worry?	1	2	3	4
23. Did you feel irritable?	1	2	3	4
24. Did you feel depressed?	1	2	3	4
25. Have you had difficulty remembering things?	1	2	3	4
26. Has your physical condition or medical treatment interfered with your family life?	1	2	3	4
27. Has your physical condition or medical treatment interfered with your social activities?	1	2	3	4
28. Has your physical condition or medical treatment caused you financial difficulties?	1	2	3	4

For the following questions please circle the number between 1 and 7 that best applies to you

29. How would you rate your overall health during the past week?

1 2 3 4 5 6 7

Very poor — Excellent

30. How would you rate your overall quality of life during the past week?

1 2 3 4 5 6 7

Very poor — Excellent

© Copyright 1995 EORTC Quality of Life Group. All rights reserved. Version 3.0

Figure 3: QLQ-C30

Table 1: QLU-C10D health state description system and translation of QLQ-C30 responses to QLU-C10D domain levels

QLU-C10D			
Domain	*Level*	*Health state descriptions*	*Translation of QLQ-C30 response levels to QLU-C10D domain levels*
Physical Functioning (PF) *	1<br>2<br><br>3<br><br>4	No trouble taking a long walk outside of the house<br>No trouble taking a short walk outside of the house, but at least a little trouble taking a long walk<br>At least a little trouble taking a short walk outside of the house, and at least a little trouble taking a long walk<br>Quite a bit or very much trouble taking a short walk outside the house	Item 2 (long walk) = 1<br>Item 3 (short walk) = 1 AND Item 2 ≥ 2<br>Item 3 = 2 AND Item 2 ≥ 2<br><br>Item 3 ≥ 3 AND Item 2 ≥ 2
Role Functioning (RF)	1<br>2<br>3<br>4	Not at all limited in pursuing work or other daily activities<br>A little limited in pursuing work or other daily activities<br>Quite a bit limited in pursuing work or other daily activities<br>Very much limited in pursuing work or other daily activities	Item 6 = 1<br>Item 6 = 2<br>Item 6 = 3<br>Item 6 = 4
Social Functioning (SF) *	1<br>2<br>3<br>4	Physical condition or medical treatment interferes **not at all** with social or family life<br>Physical condition or medical treatment interferes **a little** with social or family life<br>Physical condition or medical treatment interferes **quite a bit** with social or family life<br>Physical condition or medical treatment interferes **very much** with social or family life	Items 26 AND 27 = 1<br>Items 26 OR 27 = 2<br>Items 26 OR 27 = 3<br>Items 26 OR 27 = 4
Emotional Functioning (EF)	1<br>2<br>3<br>4	**not at all** feeling depressed<br>feeling **a little** depressed<br>feeling **quite a bit** depressed<br>feeling **very much** depressed	Item 24 = 1<br>Item 24 = 2<br>Item 24 = 3<br>Item 24 = 4
Pain (Pa)	1<br>2<br>3<br>4	**no** pain<br>**a little** pain<br>**quite a bit** pain<br>**very much** pain	Item 9 = 1<br>Item 9 = 2<br>Item 9 = 3<br>Item 9 = 4
Fatigue (Fa)	1<br>2<br>3<br>4	**not at all** tired<br>**a little** pain<br>**quite a bit** pain<br>**very much** pain	Item 18 = 1<br>Item 18 = 2<br>Item 18 = 3<br>Item 18 = 4
Sleep disturbance (Sl)	1<br>2<br>3<br>4	**no** trouble sleeping<br>**a little** trouble sleeping<br>**quite a bit** trouble sleeping<br>**very much** trouble sleeping	Item 11 = 1<br>Item 11 = 2<br>Item 11 = 3<br>Item 11 = 4
Appetite loss (Ap)	1<br>2<br>3<br>4	**not at all** lacking appetite<br>**a little** lacking appetite<br>**quite a bit** lacking appetite<br>**very much** lacking appetite	Item 13 = 1<br>Item 13 = 2<br>Item 13 = 3<br>Item 13 = 4
Nausea (Na)	1<br>2<br>3<br>4	**not at all** feeling nauseated<br>**a little** feeling nauseated<br>**quite a bit** feeling nauseated<br>**very much** feeling nauseated	Item 14 = 1<br>Item 14 = 2<br>Item 14 = 3<br>Item 14 = 4
Bowel problems (Bo)	1<br>2<br>3<br>4	**no** constipation or diarrhoea<br>**a little** constipation or diarrhoea<br>**quite a bit** constipation or diarrhoea<br>**very much** constipation or diarrhoea	Items 16 AND 17 = 1<br>Items 16 OR 17 = 2<br>Items 16 OR 17 = 3<br>Items 16 OR 17 = 4
* In the rare case of responses on PF or SF that do not follow the logical order (e.g. more severe impairment on "short walk" than on "long walk") use the more severe health state.			

3.3. EORTC QLU-C10D valuations

Eliciting health preferences using a discrete choice experiment (DCE)

EORTC QLU-C10D valuations are performed using a standardised and well-validated methodology developed by the MauCa consortium (Norman, Viney et al. 2016). Health preferences are elicited using a discrete-choice experiment (DCE). In the DCE each respondent is presented with 16 different choice sets which comprise two hypothetical health scenarios each consisting of a health state defined by the ten domains of the QLU-C10D and a survival time in that respective health state (1, 2, 5 or 10 years) (an example choice set is shown in Figure 4). Respondents have to select their preferable health scenario in each choice set. This is a forced choice, meaning they cannot progress without providing an answer. The selection of 16 choice sets each respondent is asked to complete are selected randomly out of a total of 960 which are determined by methods of optimal design theory.

Since a total of 11 attributes (10 HRQOL domains + survival time) represents quite a complex health scenario, in each choice set only five attributes are allowed to differ between the described health states in order to keep the cognitive burden at a manageable level. The order of the ten HRQOL domains is randomised for each respondent but kept constant within each individual DCE. The DCE has been thoroughly tested with regard to impact of order of attributes (Norman, Kemmler et al. 2016), graphical presentation (Norman, Viney et al. 2016), and test-retest reliability (Gamper, Holzner et al. 2018).

	Situation A	Situation B
In taking a long walk	You have a little trouble	You have a little trouble
In taking a short walk	You have a little trouble	You have a little trouble
You are limiting in pursuing your daily activities	Quite a bit	Quite a bit
Your physical condition interferes with your social or family life	Quite a bit	A little
You feel depressed	Quite a bit	Quite a bit
You have pain	A little	Very much
You feel tired	A little	A little
You have trouble sleeping	Not at all	Not at all
You lack appetite	Quite a bit	Quite a bit
You feel nauseated	A little	Quite a bit
You have constipation or diarrhoea	Not at all	Quite a bit
You will live in this health state for	2 years and then die	5 years and then die
**Which situation would you prefer?**	○	○

Figure 4: Example choice set

Standard valuation survey, respondents and sampling

A standardised valuation procedure has been put in place. Deviations from this procedure need to be approved by the Mauca Consortium, the EORTC QLD, and the EORTC HTA working group – please contact the EORTC QLD if needed.

To reflect societal health values, preferences are obtained from general population samples and are estimated separately per country. A national valuation sample comprises approximately 1,000 adult respondents (de Bekker-Grob, Donkers et al. 2015); this number was selected as it

exceeded the various rules of thumb around sample size requirements for DCEs, and also the typical range of samples used in existing health DCEs (Soekhai, de Bekker-Grob et al. 2019). Recruitment is done using online panels and the valuation survey is performed web-based, applying quota sampling for age and sex according to national census data. The DCEs are the main body of the valuation survey, which in addition includes sociodemographic and basic clinical information, the EORTC QLQ-C30, four feedback questions on the DCE, self-completion of the EQ-5D-5L (Herdman, Gudex et al. 2011), and the Kessler K-10 mental health questionnaire (Kessler, Andrews et al. 2002).

4. PROPERTIES AND APPLICATION

4.1. PSYCHOMETRIC PROPERTIES

Information on sensitivity and responsiveness - section to be written

4.2. ADMINISTRATION

The items of the EORTC QLU-C10 have to be completed by the patients themselves. It is strongly recommended/required that the entire parent instrument EORTC QLQ-C30 is administered which includes all QLU-C10D items. This allows the obtaining of the full utility of EORTC QLU-C10 as well as a full EORTC QLQ-C30 HRQOL profile. Possible modes of administration are paper-pencil based or electronic assessment (for information on electronic data capture please refer to the EORTC QLD). It is also possible to use the EORTC CAT Core measures since they likewise include all items of the QLQ-C30 (Petersen, Aaronson et al. 2018). For more information on requirements and regulations CAT administration please refer to the EORTC QLD.

Figure for overview of administration modes and required procedures

4.3. SCORING AND CONVERTING HEALTH STATES TO AN INDEX VALUE

Scoring and converting health states into an index (i.e. utility) value are performed as follows. Describe EORTC QLU-C10D health state using classification system (see Table 1):

a. Record responses to QLQ-C30 items: 2, 3, 6, 9, 11, 13, 14, 16, 17, 18, 24, and 27
b. Check validity: only one response per item and no missing item scores.
c. Describe EORTC QLU-C10D health state by score responses using Table 1. It is customary to describe a specific health state by listing the single domain scores in the order in which the domains appear in the instrument. For the QLU-C10D the order of domains is as listed in Table 1 (PF, RF, SF, EF, Pa, Fa, Sl, Ap, Na, Bo (see scoring example in section 4.5).

1. Transform QLU-C10D health state into an index value by attaching the domain scores with the respective weights: the scoring algorithm attaches a weight to each level of each dimensions and subtracts the sum of the weights from 1 (i.e. from "full health"):

$$\text{QLU-C10D utility} = 1- (\text{response}_{PF}* \text{responseweight}_{PF} + \text{response}_{RF}* \text{responseweight}_{RF} + \text{response}_{SF}* \text{responseweight}_{SF} + \text{response}_{EF}* \text{responseweight}_{EF} + \text{response}_{Pa}* \text{responseweight}_{Pa} + \text{response}_{Fa}* \text{responseweight}_{Fa} + \text{response}_{Sl}* \text{responseweight}_{Sl} + \text{response}_{Ap}* \text{responseweight}_{Ap} + \text{response}_{Na}* \text{responseweight}_{Na} + \text{response}_{Bo}*$$

Section 4.5 provides a detailed example utility calculation from EORTC QLQ-C30 scores.

Appendix A provides scoring templates for SPSS. Country specific utility weights need to be inserted and can be obtained from the publication of the respective national value set/tariff or directly from the EORTC QLD (see Table X)

Table 2: List of value sets for the EORTC QLU-C10D

4.4. ORGANISING QLU-C10D DATA

We suggest entering QLU-C10D data into a database as follows (see Table 1 for translation of QLQ-C30 levels to QLU-C10D domain levels; NOTE! PF, SF, and Bo consist of two items each). To minimise error, where possible we suggest using scoring templates (SPSS, STATA, SAS) provided in the appendix for QLU-C10D scoring directly from QLQ-C30 data.

**VARIABLE NAME**	ID	COUNTRY	TARIFF (country, year)	PF	RF	SF	EF	PA	Fa	Sl	Ap	Na	Bo
**VARIABLE DESCRIPTION**	Patient ID			1-no problems<br>2-a little problems<br>3-moderate problems<br>4-severe problems	1-no problems<br>2-a little problems<br>3-moderate problems<br>4-severe problems	1-no problems<br>2-a little problems<br>3-moderate problems<br>4-severe problems	1-no problems<br>2-a little problems<br>3-moderate problems<br>4-severe problems	1-no problems<br>2-a little problems<br>3-moderate problems<br>4-severe problems	1-no problems<br>2-a little problems<br>3-moderate problems<br>4-severe problems	1-no problems<br>2-a little problems<br>3-moderate problems<br>4-severe problems	1-no problems<br>2-a little problems<br>3-moderate problems<br>4-severe problems	1-no problems<br>2-a little problems<br>3-moderate problems<br>4-severe problems	1-no problems<br>2-a little problems<br>3-moderate problems<br>4-severe problems

4.5.QLU-C10D scoring example

1. EXAMPLE: Record responses to QLQ-C30 items: 2, 3, 6, 9, 11, 13, 14, 16, 17, 18, 24, and 27 score according to Table 1

EORTC QLQ-C30 (version 3)

We are interested in some things about you and your health. Please answer all of the questions yourself by circling the number that best applies to you. There are no "right" or "wrong" answers. The information that you provide will remain strictly confidential.

Please fill in your initials:
Your birthdate (Day, Month, Year):
Today's date (Day, Month, Year):

	Not at All	A Little	Quite a Bit	Very Much	
1. Do you have any trouble doing strenuous activities, like carrying a heavy shopping bag or a suitcase?	1	2	3	④	
2. Do you have any trouble taking a long walk?	1	2	③	4	PF ➡ 3
3. Do you have any trouble taking a short walk outside of the house?	1	②	3	4	
4. Do you need to stay in bed or a chair during the day?	1	②	3	4	
5. Do you need help with eating, dressing, washing yourself or using the toilet?	①	2	3	4	

During the past week:

	Not at All	A Little	Quite a Bit	Very Much	
6. Were you limited in doing either your work or other daily activities?	①	2	3	4	RF ➡ 1
7. Were you limited in pursuing your hobbies or other leisure time activities?	①	2	3	4	
8. Were you short of breath?	1	2	③	4	
9. Have you had pain?	1	②	3	4	PA ➡ 2
10. Did you need to rest?	1	2	3	④	
11. Have you had trouble sleeping?	1	2	③	4	SL ➡ 3
12. Have you felt weak?	①	2	3	4	
13. Have you lacked appetite?	1	2	3	④	AP ➡ 4
14. Have you felt nauseated?	1	②	3	4	NA ➡ 2
15. Have you vomited?	①	2	3	4	
16. Have you been constipated?	①	2	3	4	BO ➡ 3

Please go on to the next page

During the past week:

	Not at All	A Little	Quite a Bit	Very Much	
17. Have you had diarrhea?	1	2	③	4	BO* ➡ 3
18. Were you tired?	①	2	3	4	FA ➡ 1
19. Did pain interfere with your daily activities?	1	②	3	4	
20. Have you had difficulty in concentrating on things, like reading a newspaper or watching television?	①	2	3	4	
21. Did you feel tense?	1	②	3	4	
22. Did you worry?	1	2	③	4	
23. Did you feel irritable?	1	②	3	4	
24. Did you feel depressed?	1	2	③	4	EF ➡ 3
25. Have you had difficulty remembering things?	①	2	3	4	
26. Has your physical condition or medical treatment interfered with your family life?	1	②	3	4	SF* ➡ 2
27. Has your physical condition or medical treatment interfered with your social activities?	1	②	3	4	
28. Has your physical condition or medical treatment caused you financial difficulties?	1	2	③	4	

For the following questions please circle the number between 1 and 7 that best applies to you

29. How would you rate your overall health during the past week?

1 2 3 4 5 6 7

Very poor — Excellent

30. How would you rate your overall quality of life during the past week?

1 2 3 4 5 6 7

Very poor — Excellent

© Copyright 1995 EORTC Quality of Life Group. All rights reserved. Version 3.0

2. **EXAMPLE: Describe EORTC QLU-C10D health state: domains in order as in Table 1 (PF, RF, SF, PA, Fa, Sl, Ap, Na, Bo): 3123213423**

3. **EXAMPLE: Transform EORTC QLU-C10D health state into an index value**

Attach weights (here Australian utility weights from King et al. 2018 PharmacoEconomics **36**, 225–238) to QLU-C10D health state=3123213423

Dimension	Level	Utility decrement, w_{dl} (95% CI)
Physical functioning	1	0
	2	– 0.081 (– 0.051 to – 0.110)
	3	– 0.151 (– 0.120 to – 0.182)
	4	– 0.250 (– 0.220 to – 0.280)
Role functioning	1	0
	2	– 0.024 (0.001 to – 0.049)
	3	– 0.090 (– 0.066 to – 0.114)
	4	– 0.139 (– 0.117 to – 0.161)
Social functioning	1	0
	2	0.000 (0.024 to – 0.025)
	3	– 0.064 (– 0.040 to – 0.089)
	4	– 0.091 (– 0.070 to – 0.112)
Emotional functioning	1	0
	2	– 0.020 (0.003 to – 0.043)
	3	– 0.066 (– 0.041 to – 0.091)
	4	– 0.133 (– 0.112 to – 0.155)
Pain	1	0
	2	– 0.053 (– 0.029 to – 0.078)
	3	– 0.129 (– 0.105 to – 0.153)
	4	– 0.155 (– 0.133 to – 0.177)
Fatigue	1	0
	2	– 0.023 (– 0.001 to – 0.045)
	3	– 0.029 (– 0.006 to – 0.053)
	4	– 0.037 (– 0.016 to – 0.058)
Sleep	1	0
	2	– 0.033 (– 0.012 to – 0.054)
	3	– 0.039 (– 0.020 to – 0.059)
	4	– 0.039 (– 0.020 to – 0.059)
Appetite	1	0
	2	– 0.028 (– 0.006 to – 0.049)
	3	– 0.050 (– 0.030 to – 0.070)
	4	– 0.050 (– 0.030 to – 0.070)
Nausea	1	0
	2	– 0.047 (– 0.025 to – 0.070)
	3	– 0.068 (– 0.044 to – 0.092)
	4	– 0.107 (– 0.086 to – 0.127)
Bowel problems	1	0
	2	– 0.047 (– 0.025 to – 0.068)
	3	– 0.078 (– 0.054 to – 0.102)
	4	– 0.094 (– 0.073 to – 0.115)

Table from King et al. 2018 PharmacoEconomics **36**, 225–238

QLU-C10D utility for health state 3123213423 = 1- (0.151+ 0 + 0 + 0.066 + 0.053 + 0 + 0.039 + 0.050 + 0.047+ 0.078) = **0.516**

4. EXAMPLE: Organising QLU-C10 data

ID	COUNTRY	TARIFF (country, year)	PF	RF	SF	EF	PA	Fa	Sl	Ap	Na	Bo	QLUC10D_utility
1	AUS	Australia, 2018	3	1	2	3	2	1	3	4	2	3	0.516

5. PRESENTATION OF RESULTS

5.1. QLU-C10D index score

The most important target value for health economic analyses evaluation is the QLU-C10D index value. The index can be presented as mean and standard deviation or, in case of skewed data or very small sample sizes, as median and interquartile range. It is recommended that information is provided on the extent of ceiling/floor effects in the specific sample(s). Figure x provides an example on how to present QLU-C10D index values.

5.2. Health profiles

Please note that QLU-C10D domains are not equivalent to the QLQ-C30 domains as they do not all contain all items of the respective scale. Therefore, single domain scores are calculated from all QLQ-C30 items and reported as described in the QLQ-C30 scoring manual (Fayers, Aaronson et al. 2001). The manual includes scoring codes for SAS, SPSS, and STATA and can be downloaded for free from the EORTC QLG website (https://qol.eortc.org/manuals/). QLQ-C30 domains are always scaled from 0–100. If not otherwise specified by a hypothesis, preferably all QLQ-C30 scores are reported.

Figure y shows an example of how health profiles may look like.

6. AVAILABLE VALUE SETS AND FORMATS

In principle, the QLU-C10D can be administered in any language the QLQ-C30 has been translated to (for current status see QLG website www.qlg.eortc.be) but be aware that not all countries for which translations are available have developed accompanying value sets. It is common practice with other PBMs to apply a national value set to data collected in another country – very often in an international study setting this is also inevitable. Usually the perspective of the country in which the respective health care decision will/should be made is taken. However, so far there is no hard evidence with regard to the impact of applying a value set on “non-national” data. There is some indication that potentially as a result of differences in translations and response behaviours across countries/cultures, the respective national value set is the best choice for the data (Kemmler, Gamper et al. 2019). Since there is an ongoing discussion who’s preferences should be used, i.e. whether societal values or patient values are more appropriate and informative the EORTC QLG also will provide a patient value set.
Figure 5 shows an overview of completed and ongoing valuations and Table 2 provides a list of currently available value sets.

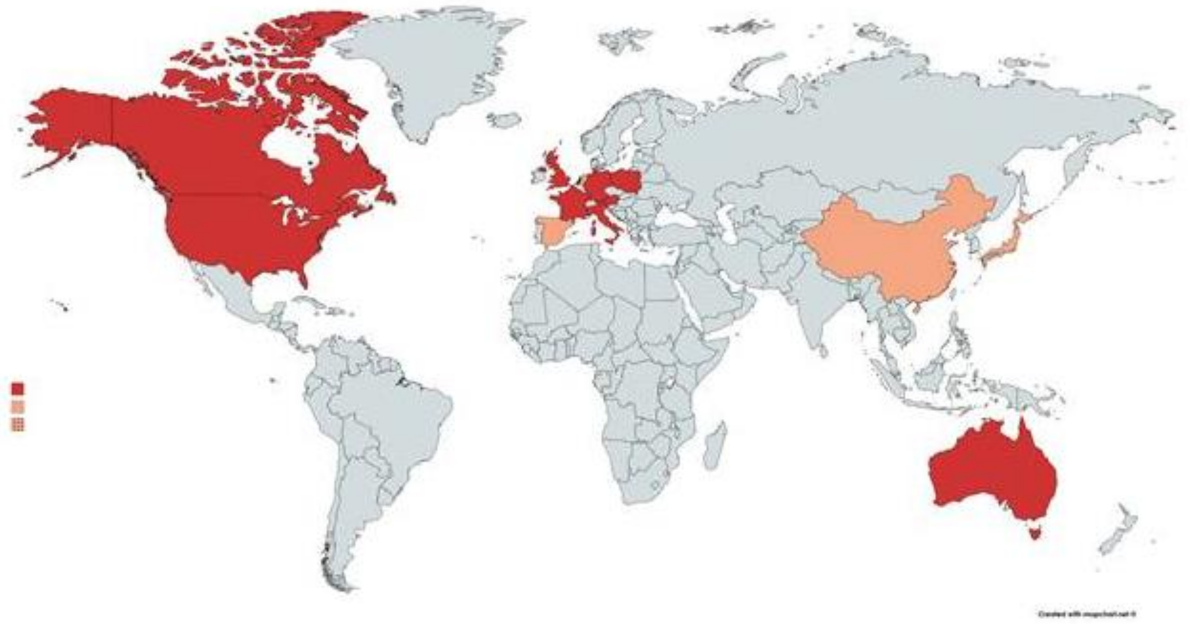

Figure 5: Overview of completed and ongoing valuations

#	Country	Year	Reference	Reference Link	Syntax / Code Link
1	Australia	2018	King MT, Viney R, Pickard AS, Rowen D, Aaronson NK, Brazier JE, Cella D, Costa DSJ, Fayers PM, Kemmler G, McTaggart-Cowan H, Mercieca-Bebber R, Peacock S, Street DJ, Young TA, Norman R. (2018) Australian utility weights for the EORTC QLU-C10D, a multi-attribute utility instrument derived from the cancer-specific quality of life questionnaire, EORTC QLQ-C30. *Pharmacoeconomics* 36(2):225-238	https://link.springer.com/article/10.1007/s40273-017-0582-5 https://doi.org/10.1007/s40273-017-0582-5	
2	Austria	2020	Gamper EM, King MT, Norman R, Efficace F, Cottone F, Holzner B, Kemmler GW. (2020) EORTC QLU-C10D value sets for Austria, Italy, and Poland. *Quality of Life Research* 29(9):2485-2495	https://link.springer.com/article/10.1007/s11136-020-02536-z https://pubmed.ncbi.nlm.nih.gov/32458409/ https://doi.org/10.1007/s11136-020-02536-z	
3	Canada	2019	McTaggart-Cowan H, King MT, Norman R, Costa D, Pickard AS, Regier D, Viney R, Peacock S. (2019) The EORTC QLU-C10D: The Canadian Valuation Study and Algorithm to Derive Cancer-Specific Utilities From the EORTC QLQ-C30. *MDM Policy and Practice* 4(1):2381468319842532	https://journals.sagepub.com/doi/abs/10.1177/2381468319842532 https://pubmed.ncbi.nlm.nih.gov/31245606/	
4	France	2020	Nerich V, Gamper EM, Norman R, King M, Holzner B, Kemmler G. (2021) French value set of the QLU-C10D, a Cancer-Specific Utility Measure Derived from the QLQ-C30. *Applied Health Economics and Health Policy*, 19(2), 191-202.	https://link.springer.com/article/10.1007/s40258-020-00598-1 https://pubmed.ncbi.nlm.nih.gov/32537694/	
5	Germany	2019	Kemmler G, Gamper E, Nerich V, Norman R, Viney R, Holzner B, King MT. (2019) German value sets for the EORTC QLU-C10D, a cancer-specific utility instrument based on the EORTC QLQ-C30. *Quality of Life Research* 28(12):3197-3211	https://link.springer.com/article/10.1007/s11136-019-02283-w https://pubmed.ncbi.nlm.nih.gov/31485913/	
6	Italy	2020	Gamper EM, King MT, Norman R, Efficace F, Cottone F, Holzner B, Kemmler GW. (2020) EORTC QLU-C10D value sets for Austria, Italy, and Poland. *Quality of Life Research* 29(9):2485-2495	https://link.springer.com/article/10.1007/s11136-020-02536-z https://pubmed.ncbi.nlm.nih.gov/32458409/ https://doi.org/10.1007/s11136-020-02536-z	
7	Netherlands	2021	Jansen F, Verdonck-de Leeuw IM, Gamper E, Norman R, Holzner B, King M, Kemmler G. (2021) Dutch utility weights for the EORTC cancer-specific utility instrument: the Dutch EORTC QLU-C10D. *Quality of Life Research*, 30(7), 2009-2019 (2021).	https://link.springer.com/article/10.1007/s11136-021-02767-8 https://pubmed.ncbi.nlm.nih.gov/33512653/	

8	Poland	2020	Gamper EM, King MT, Norman R, Efficace F, Cottone F, Holzner B, Kemmler GW. (2020) EORTC QLU-C10D value sets for Austria, Italy, and Poland. *Quality of Life Research* 29(9):2485-2495	https://link.springer.com/article/10.1007/s11136-020-02536-z https://pubmed.ncbi.nlm.nih.gov/32458409/ https://doi.org/10.1007/s11136-020-02536-z	
9	UK	2019	Norman R, Mercieca-Bebber R, Brazier JE, Cella D, Pickard AS, Rowen D, Street DJ, Viney R, King MT. (2019) UK utility weights for the EORTC QLU-C10D. *Health Economics* 28(12):1385-1401	https://doi.org/10.1002/hec.3950. PMID: 31482619.	
10	USA	2021	Revicki D, King MT, Viney R, Pickard AS, Mercieca-Bebber R, Shaw JW, Norman R. (2021) United States utility algorithm for the EORTC QLU-C10D, a multi-attribute utility instrument based on a cancer-specific quality of life instrument. *Medical Decision Making*, 41(4), 485-501.	https://pubmed.ncbi.nlm.nih.gov/29270835/	

Value sets are continuously developed and their current status can be seen on the QLG website. In case no country-specific value set is available, the analyst has to consider which country with an existing value set is the closest in terms of culture, healthcare setting and language. Where there is uncertainty on that point, we recommend that the economic evaluation includes a sensitivity analysis exploring the relationship between choice of valuation algorithm and the results of the economic evaluation.

7. QLU-C10D NORMATIVE DATA

Normative data support the applicability and interpretability of PBMs by enabling the normative comparisons across specific populations or patient groups [7] and by providing estimates for adjustments for sex and age in health economic evaluations in order to avoid confounding by these variables when comparing groups with different age- and sex-distributions. Normative scores may provide an adequate baseline in economic modelling and a comparator for survivorship studies. Therefore the provision of general population utility norms of PBM is suggested [8–10]. General population utility norms allow the comparison of HSUVs between cancer patients and a comparative group reflecting a reality, i.e. a population necessarily containing people with various (chronic) diseases, rather than a hypothetically completely healthy population. This is because in a best case treatment scenario a cancer patient population will not be returning to a perfect state of health but will still include health impairments with the same prevalence as the general population. Additionally, normative scores can facilitate comparisons across countries, regions and cultures [11], enabling the detection of health inequities in subgroups of the population [10]. General population utility norms are currently available for commonly applied multi-attribute utility instruments (MAUIs), such as the EQ-5D [12] and the SF-6D [9,13].

To support the interpretability of HSUVs obtained by the EORTC QLU-C10D, a first set of general population norms for the countries of Canada, France, Germany, Italy, Poland and the UK, for which EORTC QLU-C10D value sets have recently become available [14–18], has been provided (reference – submitted).

8. CAN I CONVERT UTILITIES OBTAINED WITH ANOTHER INSTRUMENT INTO QLU-C10D UTILITIES?

Mapping from the QLQ-C30 into the EQ-5D will not result in the same utility scores as using the QLU-C10D and serves a different purpose than measuring QLU-C10D utilities (e.g. in case EQ-5D utilities are required and cannot be obtained directly). Available mapping algorithms for the QLQ-C30 have not been provided or approved by the EORTC QLG and resulting values cannot be considered equivalent to QLU-C10D utilities.

9. HOW TO OBTAIN THE QLU-C10D AND TERMS OF USE

The copyright for the QLU-C10D health description system is with the EORTC with all rights reserved. Written prior consent of the EORTC QLD is required for the use and the administration of the QLQ-C30; there is no separate QLU-C10D licence. There is no charge for academic users (see Appendix X how to contact the QLD). The QLQ-C30 may be obtained from the EORTC QLG website (https://qol.eortc.org/questionnaires/) or by directly contacting the EORTC QLD (see….).

10. HOW TO REFER TO THE QLU-C10D IN PUBLICATIONS

When using the QLU-C10D please provide the reference to the development of the QLU-C10D health state classification system (QLU-C10D: a health state classification system for a multi-attribute utility measure based on the EORTC **QLQ-C30**. King MT, Costa DS, Aaronson NK, Brazier JE, Cella DF, Fayers PM, Grimison P, Janda M, Kemmler G, Norman R, Pickard AS, Rowen D, Velikova G, Young TA, Viney R. Qual Life Res. 2016 Mar;25(3):625-36. doi: 10.1007/s11136-015-1217-y. Epub 2016 Jan 20. PMID: 26790428) as well as the reference of the respective value set.

11. WHERE TO FIND INFORMATION ABOUT CURRENTLY ONGOING EORTC QLU-C10 STUDIES

Documents containing the scoring algorithms, information on the valuation studies, tables of values for all health states can be obtained from the EORTC QLD (web address).

12. HOW TO DEVELOP ADDITIONAL EORTC QLU-C10D VALUE SETS

The EORTC QLG and the EORTC QLD encourage and support the development of additional national QLU-C10D value sets. With regard to already ongoing valuations please refer to the EORTC website/the EORTC QLD. QLU-C10D valuations in additional countries may be performed by researchers/research groups outside the EORTC; in order to create official EORTC values sets they need to comply with the standardised methodology that is in place. The EORTC QLD and the Mauca-Consortium need to approve the valuation protocol. Please contact the EORTC QLD and/or the Mauca-consortium if you are interested in developing EORTC QLU-C10D value sets (contact details).

References

Bharmal, M. and Thomas, J. (2006). "Comparing the EQ-5D and the SF-6D Descriptive Systems to Assess Their Ceiling Effects in the US General Population." Value in health : the journal of the International Society for Pharmacoeconomics and Outcomes Research **9**(4).

Bottomley, A., Pe, M., Sloan, J., Basch, E., Bonnetain, F., Calvert, M., et al. (2016). "Analysing data from patient-reported outcome and quality of life endpoints for cancer clinical trials: a start in setting international standards." The Lancet. Oncology **17**(11).

Brazier, J., Roberts, J. and Deverill, M. (2002). "The estimation of a preference-based measure of health from the SF-36." J Health Econ **21**(2): 271-292.

Brazier, J., Roberts, J., Tsuchiya, A. and Busschbach, J. (2004). "A comparison of the EQ-5D and SF-6D across seven patient groups." Health Economics **13**(9): 873-884.

Brazier, J. E. and Tsuchiya, A. (2010). "Preference-based condition-specific measures of health: what happens to cross programme comparability?" Health Econ. **19**(2): 125-129.

Conner-Spady, B. L., Marshall, D. A., Bohm, E., Dunbar, M. J., Loucks, L., Al Khudairy, A., et al. (2015). "Reliability and validity of the EQ-5D-5L compared to the EQ-5D-3L in patients with osteoarthritis referred for hip and knee replacement." Qual Life Res **24**(7): 1775-1784.

de Bekker-Grob, E., Donkers, B., Jonker, M. and Stolk, E. (2015). "Sample Size Requirements for Discrete-Choice Experiments in Healthcare: a Practical Guide " Patient **8**(5): 373-384.

Dolan, P. (1997). "Modeling valuations for EuroQol health states." Med Care **35**(11): 1095-1108.

Drummond, M. (2005). Methods for the Economic Evaluation of Health Care Programmes. Oxford, Oxford University Press.

Färkkilä, N., Torvinen, S., Roine, R., Sintonen, H., Hänninen, J., Taari, K., et al. (2014). "Health-related quality of life among breast, prostate, and colorectal cancer patients with end-stage disease." Qual Life Res **23**(4): 1387-1394.

Fayers, P., Aaronson, N., Bjordal, K., Groenvold, M., Curran, D., Bottomley, A., et al. (2001). The EORTC QLQ-C30 Scoring Manual (3rdEdition). P. b. E. O. f. R. a. T. o. Cancer. Brussels.
Gamper, E. M., Holzner, B., King, M. T., Norman, R., Viney, R., Nerich, V., et al. (2018). "Test-Retest Reliability of Discrete Choice Experiment for Valuations of QLU-C10D Health States." Value Health **21**(8): 958-966.

Grootendorst, P., Feeny, D. and Furlong, W. (2000). "Health Utilities Index Mark 3: evidence of construct validity for stroke and arthritis in a population health survey." Medical care **38**(3).

Herdman, M., Gudex, C., Lloyd, A., Janssen, M., Kind, P., Parkin, D., et al. (2011). "Development and preliminary testing of the new five-level version of EQ-5D (EQ-5D-5L)." Qual Life Res **20**(10): 1727-1736.

Horsman, J., Furlong, W., Feeny, D. and Torrance, G. (2003). "The Health Utilities Index (HUI®): concepts, measurement properties and applications." Helath Qual Life Outcomes **1**(54).

Janssen, M. F., Pickard, A. S., Golicki, D., Gudex, C., Niewada, M., Scalone, L., et al. (2013). "Measurement properties of the EQ-5D-5L compared to the EQ-5D-3L across eight patient groups: a multi-country study." Qual Life Res **22**(7): 1717-1727.

Kemmler, G., Gamper, E., Nerich, V., Norman, R., Viney, R., Holzner, B., et al. (2019). "German value sets for the EORTC QLU-C10D, a cancer-specific utility instrument based on the EORTC QLQ-C30." Quality of Life Research **28**(12): 3197-3211.

Kessler, R. C., Andrews, G., Colpe, L. J., Hiripi, E., Mroczek, D. K., Normand, S. L., et al. (2002). "Short screening scales to monitor population prevalences and trends in non-specific psychological distress." Psychol Med **32**(6): 959-976.

Krahn, M., Bremner, K. E., Tomlinson, G., Ritvo, P., Irvine, J. and Naglie, G. (2007). "Responsiveness of disease-specific and generic utility instruments in prostate cancer patients." Qual Life Res **16**(3): 509-522.

Norman, R., Kemmler, G., Viney, R., Pickard, A. S., Gamper, E., Holzner, B., et al. (2016). "Order of Presentation of Dimensions Does Not Systematically Bias Utility Weights from a Discrete Choice Experiment." Value Health **19**(8): 1033-1038.

Norman, R., Viney, R., Aaronson, N. K., Brazier, J. E., Cella, D., Costa, D. S., et al. (2016). "Using a discrete choice experiment to value the QLU-C10D: feasibility and sensitivity to presentation format." Qual Life Res **25**(3): 637-649.

Petersen, M., Aaronson, N., Arraras, J., Chie, W., Conroy, T., Costantini, A., et al. (2018). "The EORTC CAT Core-The computer adaptive version of the EORTC QLQ-C30 questionnaire." European journal of cancer (Oxford, England : 1990) **100**.

Pickard, A., Ray, S., Ganguli, A. and Cella, D. (2012). "Comparison of FACT- and EQ-5D-based utility scores in cancer." Value in health : the journal of the International Society for Pharmacoeconomics and Outcomes Research **15**(2).

Rowen, D., Brazier, J., Ara, R. and Azzabi Zouraq, I. (2017). "The Role of Condition-Specific Preference-Based Measures in Health Technology Assessment." PharmacoEconomics **35**(S1): 33-41.

Rowen, D., Young, T., Brazier, J. and Gaugris, S. (2012). "Comparison of generic, condition-specific, and mapped health state utility values for multiple myeloma cancer." Value Health **15**(8): 1059-1068.

Rowen, D., Zouraq, I. A., Chevrou-Severac, H. and Hout, B. v. (2017). "International Regulations and Recommendations for Utility Data for Health Technology Assessment." PharmacoEconomics **35**(1): 11-19.

Sintonen, H. (2001). "The 15D instrument of health-related quality of life: properties and applications." Annals of medicine **33**(5).

Soekhai, V., de Bekker-Grob, E., Ellis, A. and Vass, C. (2019). "Discrete Choice Experiments in Health Economics: Past, Present and Future " Pharmacoeconomics **37**(2): 201-226.

Sullivan, P. W., Lawrence, W. F. and Ghushchyan, V. (2005). "A national catalog of preference-based scores for chronic conditions in the United States." Med Care **43**(7): 736-749.

The EuroQol Group (1990). "EuroQol--a new facility for the measurement of health-related quality of life." Health Policy **16**(3): 199-208.

van Dongen-Leunis, A., Redekop, W. and Uyl-de Groot, C. (2016). "Which Questionnaire Should Be Used to Measure Quality-of-Life Utilities in Patients With Acute Leukemia? An Evaluation of the Validity and Interpretability of the EQ-5D-5L and Preference-Based Questionnaires Derived From the

EORTC QLQ-C30." Value in health : the journal of the International Society for Pharmacoeconomics and Outcomes Research **19**(6).

Versteegh, M. M., Leunis, A., Uyl-deGroot, C. Y. and Stolk, E. A. (2012). "Condition-specific preference-based measures: benefit or burden? ." Value Health **15**(3): 504-513.

Wiebe, S., Guyatt, G., Weaver, B., Matijevic, S. and Sidwell, C. (2003). "Comparative responsiveness of generic and specific quality-of-life instruments." J Clin Epidemiol **56**(1): 52-60.

Appendix A

SPSS codes for converting EORTC QLQ-C30 data into QLU-C10D utility scores. For value sets not provided here please refer to the respective publication or contact the EORTC QLD for assistance.

QLU-C10D scoring algorithm for POLAND

```
*******************************************************************************
*******************************************************************************
*This code is written for SPSS users.
*Author: Eva Gamper; eva-maria.gamper@i-med.ac.at
*Assumption: For this codes to work, it is assumed that the EORTC QLQ-C30 code
* is set up as thirty columns, labelled qlq1-qlq30 (in the order as given in the questionnaire),
*each of which can take one of four values 1-4, where 1 = "Not at all", 2 = "A little",
* 3 = "Quite a  bit" and 4 = "Very much". To derive the QLU-C10D, we only need 13 of these
* Seven of QLUC10D items are single items from the EORTC QLQ-C30, and three
* (pf, sf, bo) are composite which are combined.
*******************************************************************************
*******************************************************************************
**QLU-C10D scoring algorithm for POLAND
**value set form: EORTC QLU-C10D value sets for Austria, Italy, and Poland. Gamper EM, King
**MT, Norman R, Efficace F, Cottone F, Holzner B, Kemmler G; Qual Life Res. 2020
**Sep;29(9):2485-2495.
*******************************************************************************

IF (qlq2=1) PF_u = 0 .
IF (qlq2>1) PF_u = 0.064  .
IF (qlq3>1) PF_u = 0.149  .
IF (qlq3>2) PF_u = 0.272  .
EXECUTE.

IF (qlq6=1) RF_u = 0 .
IF (qlq6=2) RF_u = 0.070   .
IF (qlq6=3) RF_u = 0.139  .
IF (qlq6=4) RF_u = 0.196  .
EXECUTE.

IF (qlq26=1 & qlq27=1) SF_u = 0 .
IF (qlq26=2 | qlq27=2) SF_u  = 0.000 .
IF (qlq26=3 | qlq27=3) SF_u  = 0.008  .
IF (qlq26=4 | qlq27=4) SF_u  = 0.033  .
EXECUTE.

IF (qlq24=1) EF_u = 0 .
IF (qlq24=2) EF_u = 0.004.
IF (qlq24=3) EF_u = 0.020 .
```

```
IF (qlq24=4) EF_u = 0.034  .
EXECUTE.

IF (qlq9=1) Pa_u = 0 .
IF (qlq9=2) Pa_u = 0.015   .
IF (qlq9=3) Pa_u = 0.067   .
IF (qlq9=4) Pa_u = 0.125  .
EXECUTE.

IF (qlq18=1) Fa_u = 0 .
IF (qlq18=2) Fa_u = 0.012 .
IF (qlq18=3) Fa_u = 0.041 .
IF (qlq18=4) Fa_u = 0.041 .
EXECUTE.

IF (qlq11=1) Sl_u = 0 .
IF (qlq11=2) Sl_u = 0.021 .
IF (qlq11=3) Sl_u = 0.025  .
IF (qlq11=4) Sl_u = 0.038  .
EXECUTE.

IF (qlq13=1) Ap_u = 0 .
IF (qlq13=2) Ap_u = 0.016  .
IF (qlq13=3) Ap_u = 0.049  .
IF (qlq13=4) Ap_u = 0.053  .
EXECUTE.

IF (qlq14=1) Na_u = 0 .
IF (qlq14=2) Na_u = 0.037  .
IF (qlq14=3) Na_u = 0.056  .
IF (qlq14=4) Na_u = 0.084  .
EXECUTE.

IF (qlq16=1 & qlq17=1) Bo_u = 0 .
IF (qlq16=2 | qlq17=2) Bo_u = 0.034   .
IF (qlq16=3 | qlq17=3) Bo_u = 0.067   .
IF (qlq16=4 | qlq17=4) Bo_u = 0.076   .
EXECUTE.


COMPUTE QLUC10D_PL = 1- (PF_u + RF_u + SF_u + EF_u + Pa_u + Fa_u + Sl_u + Ap_u + Na_u
+ Bo_u) .
FORMATS QLUC10D_PL  (F8.3).
EXECUTE.

*****************************************************************************************
* The new variable QLUC10D_PL is a utility score where full health
*(i.e. level 1 in each of the utility levels) is scored at 1, and the minimum score
```

*(i.e. each utility level is at 4) is 0.048. These data can now be used to
* construct quality-adjusted life years (QALYs) for cost-utility analysis.
****
****

QLU-C10D scoring algorithm for the NETHERLANDS

```
************************************************************************************
************************************************************************************
*This code is written for SPSS users.
*Author: Eva Gamper; eva-maria.gamper@i-med.ac.at
*Assumption: For this codes to work, it is assumed that the EORTC QLQ-C30 code
* is set up as thirty columns, labelled qlq1-qlq30 (in the order as given in the questionnaire),
*each of which can take one of four values 1-4, where 1 = "Not at all", 2 = "A little",
* 3 = "Quite a  bit" and 4 = "Very much". To derive the QLU-C10D, we only need 13 of these
* Seven of QLUC10D items are single items from the EORTC QLQ-C30, and three
* (pf, sf, bo) are composite which are combined.
************************************************************************************
************************************************************************************
**QLU-C10D scoring algorithm for the NETHERLANDS
**value set from: Dutch utility weights for the EORTC cancer-specific utility instrument: the Dutch
**EORTC QLU-C10D. Femke Jansen, Irma M Verdonck-de Leeuw, Eva Gamper, Richard
**Norman, Bernhard Holzner, Madeleine King, Georg Kemmler. Qual Life Res. 2021
**Jul;30(7):2009-2019.  doi: 10.1007/s11136-021-02767-8. Epub 2021 Jan.9
************************************************************************************

IF (qlq2 = 1) PF_u = 0 .
IF (qlq2>= 2 AND qlq03 = 1) PF_u = 0.036  .
IF (qlq02>= 2 AND qlq03 = 2) PF_u = 0.121  .
IF (qlq02>= 2 AND qlq03 >= 3) PF_u = 0.228  .
EXECUTE.

IF (qlq06 = 1) RF_u = 0 .
IF (qlq06  = 2) RF_u = 0.015 .
IF (qlq06 = 3) RF_u = 0.110  .
IF (qlq06 = 4) RF_u = 0.149  .
EXECUTE.

IF (qlq26 = 1 AND qlq27  = 1) SF_u = 0 .
IF (qlq26 = 2 OR qlq27 = 2) SF_u = 0.003  .
IF (qlq26 = 3 OR qlq27 = 3) SF_u = 0.060  .
IF (qlq26 = 4 OR qlq27 = 4 ) SF_u = 0.103  .
EXECUTE.

IF (qlq24 = 1) EF_u = 0 .
IF (qlq24  = 2) EF_u = 0 .
IF (qlq24  = 3) EF_u = 0.  .
```

```
IF (qlq24  = 4) EF_u = 0.083  .
EXECUTE.

IF (qlq09 = 1) Pa_u = 0 .
IF (qlq09 = 2) Pa_u = 0.  .
IF (qlq09 = 3) Pa_u = 0.095  .
IF (qlq09 = 4) Pa_u = 0.242  .
EXECUTE.

IF (qlq18 = 1) Fa_ u = 0 .
IF (qlq18 = 2) Fa_ u = 0.005  .
IF (qlq18 = 3) Fa_ u = 0.005  .
IF (qlq18 = 4) Fa_ u = 0.055  .
EXECUTE.

IF (qlq11 = 1) Sl_ u = 0 .
IF (qlq11 = 2) Sl_ u = 0.051   .
IF (qlq11 = 3) Sl_ u = 0.053   .
IF (qlq11 = 4) Sl_ u = 0.053  .
EXECUTE.

IF (qlq13 = 1) Ap_ u = 0 .
IF (qlq13 = 2) Ap_ u = 0.005  .
IF (qlq13 = 3) Ap_ u = 0.035  .
IF (qlq13 = 4) Ap_ u = 0.035   .
EXECUTE.

IF (qlq14 = 1) Na_ u = 0 .
IF (qlq14 = 2) Na_ u = 0.035  .
IF (qlq14 = 3) Na_ u = 0.079  .
IF (qlq14 = 4) Na_ u = 0.107  .
EXECUTE.

IF (qlq16 = 1 AND qlq17 = 1) Bo_ u = 0 .
IF (qlq16 = 2 OR qlq17  = 2) Bo_ u = 0.038  .
IF (qlq16 = 3 OR qlq17  = 3) Bo_ u = 0.041  .
IF (qlq16 =4 OR qlq17  = 4) Bo_ u = 0.105  .
EXECUTE.


COMPUTE QLUC10D_NL = 1- (PF_u + RF_u + SF_ u + EF_u + Pa_ u + Fa_ u + Sl_ u + Ap_ u +
Na_ u + Bo_ u) .
FORMATS QLUC10D_NL(F8.3).
EXECUTE.
```
